# A systematic narrative review of extrinsic strategies to improve affective responses to exercise

**DOI:** 10.3389/fspor.2023.1186986

**Published:** 2023-07-11

**Authors:** Leighton Jones, Zachary Zenko

**Affiliations:** ^1^ Health Research Institute, Sheffield Hallam University, Sheffield, United Kingdom; ^2^ Department of Kinesiology, California State University Bakersfield, Bakersfield, CA, United States

**Keywords:** affect, affective responses, exercise, resistance exercise, enjoyment

## Abstract

**Background:**

Extrinsic strategies affect the exercise experience but fall outside the frequency, intensity, time, and type (i.e., *dose-determining*) principles. To our knowledge, no systematic review has focused on extrinsic strategies to influence the affective responses to exercise. The objective was to identify extrinsic strategies that seek to influence affective responses during exercise and other motivationally relevant variables including post-exercise momentary affective valence, remembered and forecasted pleasure, and enjoyment.

**Methods:**

For inclusion, eligible articles reported peer-reviewed original research, used acute bouts of exercise, and used a dimensional approach for measuring affective responses or measured enjoyment post-exercise. Web of Science, PubMed, and PsychINFO databases were last searched on 10th September 2021. Quality assessment was completed following the Effective Public Health Practice Project approach. Results were presented using a narrative synthesis.

**Results:**

125 studies were included with sample descriptions, study design (extrinsic strategies, mode, type, intensity, and duration), measurement details, and results summarised for each study.

**Conclusions:**

71% of studies were categorised as Weak according to the quality assessment tool with sampling practices (self-referred participants) and poor reporting of participant withdrawals/drop-outs the predominant reasons for Weak ratings. A wide variety of extrinsic strategies were reported with music, music videos, immersive virtual reality, outdoor exercise, caffeine, high-to-low pattern of exercise intensity, self-selected exercise intensity, and manipulation of self-efficacy offering promise as suitable strategies to positively change how people feel during exercise.

**Systematic Review Registration:**

https://osf.io/jbh8v/.

## 1. Introduction

Rates of physical inactivity in many areas of the world continue to cause physical and mental health issues. The negative effects of physical inactivity have long been established and there is no debate that increasing population-level physical activity would have significant and positive effects at individual and societal levels. However, Hallal et al. (1) explained that “The traditional public health approach based on evidence and exhortation has—to some extent—been unsuccessful so far” (p. 254). Consequently, new approaches to increasing physical activity might be required.

Recent increased interest in the role of affect in behavioural sciences has prompted some acknowledgement that a new “era of affectivism” is emerging (2). Within exercise psychology, there is growing awareness of the role that affective responses might play in promoting adherence (3). For example, Rhodes and Kates (4) performed a systematic review that indicated that the affective responses experienced *during* exercise, but not *after*, predicted future exercise behaviour. Recent theoretical developments such as affective reflective theory of physical activity and exercise [ART (5)], and the theory of effort minimization in physical activity [TEMPA (6)] adopt a dual-process approach to understanding physical activity behaviour. Moreover, those models include affective responses as deterministic automatic pre-cursors to behavioural decisions about exercise. Owing to the renewed focus on affect from traditional and emerging theoretical perspectives, researchers are seeking to develop strategies and interventions that can enhance affective responses to exercise. Evidence suggests that enhancing how people feel while they move may be most meaningful (4).

Physical activity is a broad term capturing “any bodily movement produced by skeletal muscles that results in energy expenditure” (7), with exercise and sport considered types of physical activity. Sport and exercise both include bodily movement, but a key difference between physical activity and exercise is the “planned, structured, and repetitive bodily movements” (7), and the distinguishing feature of sport is the competition regulated by a regulatory agency (8). This review focuses on affect enhancing strategies that have been developed in an exercise context. Exercise can be characterised by four principles: Frequency, Intensity, Time, and Type (FITT). There are numerous strategies that have been developed with a focus on these dose-determining *intrinsic* characteristics [see Jones and Zenko (9)]. In addition to those intrinsic characteristics, are *extrinsic* factors that relate to the broader exercise environment and encompass anything related to exercise that is outside of the FITT principles (i.e., factors that will not change the dose of exercise) (9). For example, the intensity (70% maxHR), time (60 min), and type (running) are intrinsic factors but whether that activity is conducted outside or inside, or with or without music would be the extrinsic factors. This review seeks to capture studies examining the role that extrinsic strategies could play in changing affective responses to exercise. Intensity of exercise is a crucial determinant of affective response [e.g., (10)] and has previously been captured in a review by Ekkekakis et al. (11). However, a review including extrinsic strategies designed to improve affective responses to exercise has not yet been conducted and would assist researchers in understanding the current state of the field and opportunities for future advancements. Because this is, to our knowledge, the first review of its kind, we take a broad approach and include any factor outside of directly changing overall workload. We also take a broad and inclusive approach regarding participant inclusion criteria; this review includes diverse populations (e.g., trained individuals, untrained individuals, older adults).

The terms core affect, moods, and emotions are often used in exercise literature to capture how people feel about their experience. However, concerns over the suitability of how these terms and concepts have been implemented to capture how people feel during and after exercise have been raised [see Ekkekakis (12)]. Suitability of the use of those terms concerns the conceptual understanding of what each of them refer to. The definition of those terms in the present review follows previously proposed descriptions [e.g., (12, 13)]. Briefly, core affect is an always present state conceptualised with an arousal and valence dimension and is the “most elementary consciously accessible affective feeling” (14). We adopt Russell's 1980 cirumplex model of affect as a conceptual model and a dimensional measurement approach based on its apparent superiority in allowing broad domain coverage of affective responses, compared to the distinct-states approach, which may allow for the measurement of distinct feeling states during exercise (e.g., calmness, vigor) but may also fail to assess all theoretically possible changes in affect [see (12)]. In-task assessments of affect appear more useful with regards to predicting future behaviour with a review article stating that “positive changes in basic affective responses during moderate intensity exercise was reliably linked to future physical activity behavior” (4). Owing to the issues with unsuitable measurement approaches in some studies, the present review seeks to capture those studies that align: (1) relevant affective phenomena, (2) appropriate conceptual models, (3) strong psychometric properties, and (4) assessment at appropriate time points. Affective outcomes measured using a dimensional approach will be prioritized (12) and the review will separately examine (a) in-task, moment-to-moment affective valence (4), (b) post-task, momentary affective valence, and (c) post-task, remembered and forecasted affective responses [e.g., remembered pleasure of the physical activity or exercise session, predicted pleasure of future physical activity or exercise sessions, or enjoyment (9)].

As interest in the affective responses to exercise grows, so do the attempts by researchers to influence those responses. One of the most heavily researched extrinsic strategies is music. A recent meta-analysis by Terry et al. (15) on the effects of music in sport and exercise included 139 studies from the past 100 years. The broad ranging review captured psychological, physiological, psychophysical, and performance outcomes that demonstrated the depth of research on this popular strategy. The effects of outdoor exercise (e.g., Green Exercise) is garnering increased interest and the systematic review by Lahart et al. (16) included 28 trials examining the physical (e.g., cortisol, heart rate) and mental health (e.g., depression) effects of green exercise. There are also other approaches that have received less attention to date but offer innovative and emerging approaches to positively influence how people feel during exercise (e.g., virtual reality, mindfulness). The present review seeks to capture the effects of these strategies in a focused way that is designed to assist researchers and practitioners specifically interested in influencing affective responses to exercise.

The purposes of this review are to identify extrinsic strategies that aim to: influence affective responses during exercise; influence post-task momentary affective valence; influence post-task remembered and forecasted pleasure; influence enjoyment. This review will take inventory of extrinsic strategies that have been used to alter the affective experience of exercise. Experimental studies that manipulate the exercise experience with the aim of influencing affective responses using extrinsic strategies are the focus of this review. Although studies that may indirectly influence intensity (e.g., by allowing people to listen to music) or involve intensity (e.g., by comparing self-selected intensity to a matched, prescribed intensity) are included in this review, studies that solely manipulate the intensity or workload of exercise are not within the scope of this review. Similarly, studies that compare types (or modes) of exercise (e.g., running compared to swimming) are not within the scope of this review.

## 2. Method

**Figure 1:**
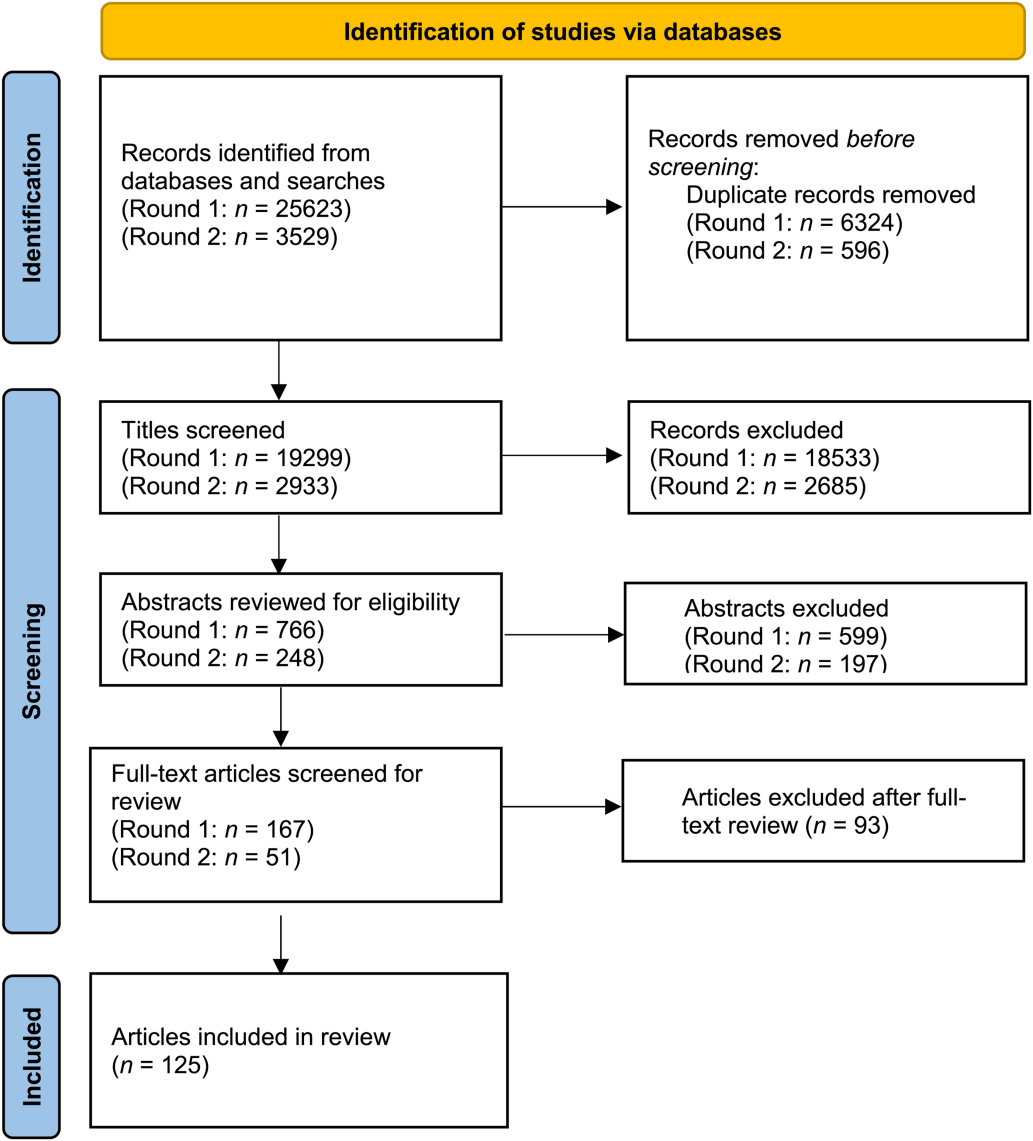
PRISMA flow diagram of search process.

This systematic review was conducted according to the Preferred Reporting Items for Systematic Reviews and Meta-Analyses (PRISMA) guidelines. The PRISMA flow diagram is presented in Figure 1.

### 2.1. Eligibility criteria

A study was considered for the review if the following eligibility criteria were met: (a) they were peer-reviewed, original research and published in the English language, (b) acute bouts of physical activity were studied (e.g., planned exercise), (c) a dimensional approach to measuring affective responses was used to assess in-task core affect, or enjoyment was measured following physical activity. In line with the aim of the review, all eligible studies included an extrinsic strategy that aimed to influence affective responses during the activity.

### 2.2. Search strategy

The search strategy was pre-registered on 7 July 2020 (https://osf.io/jbh8v/). Searches were conducted from 12 July 2020 to 10 September 2021 and included Web of Science, PubMed, and PsychINFO databases. Combinations of the following search terms were used: “exercise”, “sport”, “physical activity”, core affect”, “affective valence”, “pleasure”, “affective responses”, “feeling scale”, “enjoyment”, and “mood”. Additionally, reference lists were checked for other eligible articles.

### 2.3. Screening

Both authors collected and screened articles for eligibility and a consensus was reached in cases of disagreements. Initial screening included article titles and studies were excluded if both authors agreed the study was not suitable for inclusion based the described criteria. Authors then screened abstracts for inclusion and subsequently screened full-text articles. Data were extracted to an Excel workbook.

### 2.4. Data abstraction and analysis

Each author extracted data from half of the eligible full-text articles. To check agreement and completeness, a random sample of half of the articles were examined by the other author. Extracted data included: (a) author names, year of publication, article citation information, (b) participant characteristics, (c) study design, (d) Primary manipulation, (e) type/mode of activity, (f) affective constructs, measures, collection timepoints, (g) intentions following exercise (if applicable), (h) results. Quality assessment of individual studies was completed following the approach of the Effective Public Health Practice Project (17). Specifically, (a) selection bias, (b) design, (c) confounders, (d) blinding, (e) data collection methods, and (f) withdrawals and dropouts were evaluated. Although all studies that met inclusion criteria are included in the synthesis, they were identified as either “Strong”, “Moderate”, or “Weak”. The overall percentage of Strong, Moderate, and Weak studies are reported. This tool was selected owing to its application to many public health topics.

The extracted data are summarized and synthesised to discuss strategies for facilitating pleasant exercise. Following the guidelines by Popay et al. (18), the review focuses on (a) interpreting the literature to date and discussing the applicability of the extant literature, (b) describing the effectiveness of the previously studied strategies for enhancing the pleasure of physical activity and exercise, (c) exploring the relationships within the data that may explain study results, and (d) assessing the strength of the research evidence and generalizability to understudied populations and contexts.

## 3. Results

The number of studies included was 125 (see Figure 1). Each study was assigned one or more strategy name(s) and those strategies are discussed. The strategies have been grouped under the following headings: audio-visual; indoor and outdoor exercise; nutrition; pattern of exercise intensity; prescribed vs. self-selected exercise; mindfulness; self-efficacy; social environment; gamification and exergaming; other factors. Summary data for each study is presented in Table 1.

**Table 1:** Summary table for included studies (*n* = 125).

Article	Brief sample description	Design; factors (primary manipulation); Mode and Activity Description (Type, Intensity, Duration)	Measurement details	Summary results and conclusions	Extrinsic strategy
Addoh et al. (19)	*N* = 30; 21 females, 9 males, *M* age 21 ± 0.25 years; *M* MVPA per week 231.83 ± 57.31 min	Within-subjects; Positive affect elicitation using a video clip (drunk people falling) shown before exercise; Treadmill walking for 10 min at a self-selected pace	FS measured before and during exercise	No significant differences between priming by viewing a “humorous” video of drunk people falling and the control condition	Priming, Video
Almeida et al. (20)	*N* = 28; women; *M* age 42.4 ± 4.5 years in the 90 beats per minute group, *M* age 42.7 ± 6.6 years in the 140 beats per minute group; *M* age 41.7 ± 5.0 years in the control group	Between-subjects; Music tempo, 90 beats per minute, 140 beats per minute; Treadmill for 30 min at a self-selected intensity	FS administered during exercise	There were no effects of music tempo on pleasure	Music
Aloui et al. (21)	*N* = 9; male; *M* age 21 ± 1.1 years; physical education students	Within-subjects; self-selected music and fasting during Ramadan; 10 min warm-up followed by 5 meter multiple shuttle run test at maximum effort	Enjoyment measured with a subscale of the “Enjoyment Inventory"	Enjoyment was higher before Ramadan than during Ramadan while listening to music	Music; Fasting
Astorino et al. (22)	*N* = 16; men; 8 endurance-trained, *M* age 28 ± 6.0 years, physical activity 8.6 ± 4.4 h per week; 8 active men, *M* age 26.7 ± 5.9 years, physical activity 8.3 ± 2.1 h per week	Mixed design; Caffeine; Cycling a 10 km time trial	FS before and during exercise.	FS declined in all conditions. Lower FS in trained participants compared to active participants. Higher FS scores at some time points in the second caffeine trial compared to placebo for both groups.	Nutrition; Caffeine
Ávila-Gandía et al. (23)	*N* = 25; sex not stated; *M* age 29.6 ± 5.8 years; *M* VO_2_ max 56.0 ± 7.2 ml/kg/min; triathletes	Within-subjects; Cognitive load (high vs. low); Stationary cycling at 60% VO_2_ max for 45 min	The Self-Assessment Manikin during exercise	Pleasure was lower during high cognitive loads	Cognitive load
Backhouse et al. (24)	*N* = 15; men; *M* age 21 ± 0.5 years; *M* VO_2_ max 65.0 ± 1.2 ml/kg/min	Within-subjects; Water ingestion; Treadmill at 70% VO_2_ max for 90 min	The Activation-Deactivation Adjective Checklist administered before and after exercise. The FS administered before, during and after exercise	Pleasure increased from baseline to 60 min in the water ingestion condition. Pleasure decreased from to 80 min in no water condition. Pleasure was higher post-exercise in the water condition compared to no-water condition	Nutrition; Caffeine
Backhouse et al. (25)	*N* = 17; male; *M* age 21 ± 1 years; *M* VO_2_ max 59 ± 0.8 ml/kg/min; soccer players	Within-subjects; carbohydrate or placebo ingestion; six intermittent shuttle tests of 15-min duration each (90 min total)	The FS administered before, during, and after exercise.	There were no effects on pleasure	Nutrition
Backhouse et al. (26)	*N* = 12; males; *M* age 24 ± 1 years; *M* VO_2_ max 4.7 ± 0.2 L/min; endurance runners	Within-subjects; Caffeine; Cycle ergometer at 70% VO_2_ max for 90 min	The FS administered before, during, and after exercise	Caffeine ingestion resulted in increased pleasure	Nutrition; Caffeine
Backhouse et al. (27)	*N* = 9; males; *M* age 25 ± 2 years; *M* VO_2_ Max 64.7 ± 2.7 ml/kg/min; endurance runners	Within-subjects; Carbohydrate ingestion; Cycle ergometer 70% VO_2_ max for 120 min	The FS administered before, during, and after exercise.	Carbohydrate consumption resulted in increased pleasure	Nutrition
Baldari et al. (28)	*N* = 13; males; *M* age: 27 ± 5 years; endurance runners	Within-subjects; Transcranial direct current stimulation; Treadmill exercise increasing until maximum intensity	The FS administered at the end of each stage of exercise.	There were no effects of condition on pleasure	Other; Brain stimulation
Baños et al. (29)	*N* = 109; 62 female, 47 male; *M* age 11.86 ± 1.24 years; 33 children were overweight	Within-subjects design; Virtual Reality, internal focus, distraction focused on virtual environment; Treadmill walking for 6 min	Enjoyment was measured with an *ad-hoc* measure.	No differences in enjoyment between conditions	Virtual Reality; Distraction; Attentional association
Batista et al. (30)	*N* = 30; women; *M* age 59.3 ± 6.1 years; *M* BMI 25.7 ± 3.1 kg/m^2^	Within-subjects; Prescribed and self-selected intensity; Treadmill for 20 min	The FS administered during exercise	Self-selected intensity resulted in higher pleasure	Prescribed vs. self-selected intensity
Bigliassi et al. (31)	*N* = 24; 12 male, 12 female; *M* age 23.6 ± 3.9 years	Within-subjects; Mindfulness, audio-guided mindfulness, mindlessness; Self-paced walking for 200 meters around a park on campus	PACES at the end of each condition	Mindfulness elicited the highest enjoyment and mindlessness was lowest	Mindfulness
Bigliassi et al. (32)	*N* = 24; no sex data; *M* age 28.3 ± 5.5 years; *M* BMI 32.2 ± 12.4 kg/m^2^	Within-subjects; Audiovisual stimuli, personalised video clips (e.g., movie scenes); Recumbent cycling at a self-selected intensity for 10 min	The FS administered during exercise.	FS scores higher for audiovisual condition compared to control at 2.5 min. Also, higher in AV condition compared to control and deprivation condition at 5 min, 7.5 min, and 9.5 min.	Audio; Video
Bigliassi et al. (33)	*N* = 24; 11 women, 13 men; *M* age 23.5 ± 4.3 years	Within-subjects; Audio, podcast, music; 400 m self-paced walk on an athletic track	PACES after the task.	The music condition was more enjoyable than the podcast and control conditions, and the podcast condition was more enjoyable than the control condition.	Music; Audio
Bird et al. (34)	*N* = 24; 8 women, 16 men; *M* age 21.3 ± 1.2 years; recreationally active university students	Within-subjects; Audiovisual stimuli, music videos; Cycle ergometer at lactate threshold 20 for minutes	The FS administered before, during, and after exercise.	Pleasure was higher with music video compared to control and music-only conditions for first 14 min. There was also main effect of condition favoring music video and music-only when compared to control.	Music; Video
Bird et al. (35)	*N* = 24; 10 women, 14 men; *M* age 26.7 ± 4.1 years	Within-subjects; Music and virtual reality; 12 min of cycling, 3 min warm-up, 6 min at 10% below ventilatory threshold, 3 min warm down. Manipulation for 6 min.	The FS was administered during exercise. Enjoyment was measured using the PACES.	Pleasure was higher for the virtual reality condition compared to the control condition. Enjoyment was higher in the virtual reality and virtual reality with music conditions, compared to the control condition	Music; Virtual reality
Bird et al. (36)	*N* = 18; 9 women, 9 men; *M* age 24.17 ± 4.23 years; *M* BMI 22.98 ± 3.05 kg/m^2^; *M* VO_2_ Peak 37.07 ± 6.36 ml/kg/min	Within-subjects; Audiovisual stimuli, music, music video, 360° video; Cycle ergometer at the ventilatory threshold for 10 min	The FS administered before, during, and after exercise. Enjoyment was measured with the PACES.	The music video condition was most enjoyable compared to the control condition	Music; Video
Brick et al. (37)	*N* = 24; 13 men, *M* age 41.65 ± 11.62 years, *M* VO_2_ max 47.79 ± 5.09 ml/kg/min; 11 women, *M* age 48.08 ± 9.03 years, *M* VO_2_ max 41.28 ± 4.15 ml/kg/min; trained runners	Within-subjects; Facial expression, smiling, frowning, consciously relaxing hands and upper body; Running at 70% VO_2_ max for 6 min	The FS was administered after each block of exercise. Applied as a measure of remembered pleasure.	Condition did not make a difference in pleasure	Other; Facial expression; Attentional focus
Briki and Majed (38)	*N* = 26; females; *M* age 22.35 ± 1.76 years; Study 1	Between-subjects; Colours presented on screens, green, red, blue; Treadmill walking at a self-selected “most comfortable speed” for 5 min per condition	The Self-Assessment Manikin was administered four times during exercise	There were no effects of colour	Colours
Briki and Majed (38)	*N* = 28; females, *M* age 22.96 ± 3.51 years; study 2	Between-subjects; Colours presented on screens green, red, blue; Treadmill running at a self-selected “most comfortable speed” for 5 min per condition	The Self-Assessment Manikin was administered four times during exercise	There were no effects of colour	Colours
Brown et al. (39)	*N* = 10; 6 males, 4 females; *M* age 30 ± 3 years	Within-subjects; Mouth rinsing, pink or clear non-caloric artificially sweetened solution; treadmill running at self-selected pace (equivalent of RPE of 15) for 30 min	The FS was administered 30 s prior to each mouth rinsing and following run completion.	Rinsing with a pink solution resulted in higher FS scores	Nutrition
Brownley et al. (40)	*N* = 16; 12 women, 4 men; aged 19–28 years (no mean data provided); trained and untrained	Mixed-design; Music tempo, sedative, fast; Treadmill exercise for 10 min at low, moderate, and high intensity	The FS administered prior to the end of the 10 min bout	Higher scores for fast tempo music during low and moderate intensity exercise in untrained participants	Music; Music tempo
Calogiuri et al. (41)	*N* = 19; initial sample 7 men, 7 women; *M* age 48.5 ± 7.3 years. Additional sample 4 women, 1 man; *M* age 33.4 ± 4.0 years.	Between-subjects; Indoor vs. outdoor exercise; Cycling and circuit-style strength training, 70% VO_2_max	Enjoyment measured using a 0–10 scale	Enjoyment was higher for outdoor exercise but only for cycling, not strength training	Green exercise
Calogiuri et al. (42)	*N* = 26; 14 male, 12 female; *M* age 26 ± 8 years.	Within-subjects; Immersive virtual environment, 360-degree video and audio of a nature walk, walking in nature, sitting down with VR; Treadmill exercise and nature trail at “a comfortable pace”	Enjoyment measure using a 0–10 scale. Enjoyment measured after each condition	The walk outdoors in nature was more enjoyable than walking on a treadmill using the immersive virtual environment, and sitting with the virtual environment	Green exercise; Virtual reality
Campbell et al. (43)	*N* = 148; 107 women and 41 men; *M* age 22.25 ± 5.13 years; university students	Between-subject; Music; Walking on an indoor track at a moderate intensity for 20 min	Enjoyment was assessed with the PACES. Pleasant-unpleasant scale of the Brief mood Introspection Scale, administered before and after exercise	Pleasure increased from pre-to-post exercise in the music group only. The music group also had higher enjoyment ratings	Music
Chen et al. (44)	*N* = 12; 10 males, 2 females; *M* age 22.56 ± 5.14 years; Down's Syndrome	Within-subjects; Music; treadmill walking at 50%–80% maxHR for 24 min	Enjoyment was measured using a subscale of the intrinsic motivation inventory.	Enjoyment was not significantly different between conditions	Music
Chmelo et al. (45)	*N* = 32; females; *M* age 21 ± 1.4 years; college students	Within-subject; Mirrored environment; Resistance exercise (two sets at 60% and 100% 10-RM) on seven resistance exercises plus abdominal crunches until failure	FS and the Activation-Deactivation Adjective Checklist was administered before, during, and after exercise	There were no effects on affective outcomes between conditions	Mirrors; Social Environment
Cortis et al. (46)	*N* = 18; 10 males, *M* age 20 ± 0.8 years, *M* VO_2_ max: 47.6 ± 3.7 ml/kg/min; 8 females, *M* age 21.3 ± 2.7 years; *M* VO_2_ max 41.1 ± 4.1 ml/kg/min	Within-subjects; Order of intervals, ascending, descending, mixed pyramid; Intervals on cycle ergometer at 50%, 70%, and 100% peak power output for 18 min (excluding warm up and cool down)	Enjoyment was measured using the Exercise Enjoyment Scale	There was no difference in enjoyment between conditions	Ordering of intervals
Cox et al. (47)	*N* = 23; 19 female, 3 male, and 1 “other gender”; age 19.26 ± 1.14 years; university students	Within-subjects; Mindfulness; walking at 65% HRR for 10 min	The FS was administered before, during, and after exercise. In-task measurements were averaged. Enjoyment was measured using the PACES.	In-task pleasure was higher in the mindfulness condition compared to control, and enjoyment was greater in the mindfulness condition	Mindfulness
Cox et al. (48)	*N* = 31; females; *M* age 28.6 ± 9.9 years; low active	Within-subjects; Mindfulness, music; Treadmill walking at a self-selected intensity for 20 min	The FS was administered during exercise; Enjoyment was measured using the Interest/Enjoyment subscale of the Intrinsic Motivation Inventory, Remembered and forecasted pleasure were measured using the Empirical Valence Scale (49) (EVS) and a visual analogue scale (VAS), respectively	There were no effects on FS scores. Forecasted pleasure was higher in the mindfulness condition and music condition compared to control; remembered pleasure was highest in the music condition compared to the control condition but not compared to the mindfulness condition; enjoyment was highest in the music condition compared to the control condition but not compared to the mindfulness condition	Mindfulness; Music
Cox et al. (50)	*N* = 62; female; *M* age 23.89 ± 6.86 years; college students	Between-subjects; Mindfulness, being mindfully present, changing appearance, yoga poses; Yoga for 45 min	The FS was administered before and after exercise. Remembered pleasure measured with VAS, forecasted pleasure measured with EVS	Mindfulness condition greatest increase in FS compared to appearance condition. Remembered pleasure lower in appearance condition compared to mindfulness and yoga poses. Forecast pleasure lower for appearance compared to mindfulness and yoga poses	Mindfulness
Crust et al. (51)	*N* = 83; 27 men, 56 women; Urban green walkers *M* age 64.1 ± 8.6 years; Countryside walkers *M* age 61.3 ± 9.9 years	Between-subjects; Urban green environment or countryside walk; Walking between 60 and 90 min	Enjoyment was measured with the PACES	Enjoyment was higher for countryside walking compared to the urban green environment	Green exercise
Dasilva et al. (52)	*N* = 34; 17 men, *M* age 24 ± 3.3 years; 17 women; *M* age 22.5 ± 2.6 years	Within-subjects; Indoor and outdoor exercise, treadmill, outdoor track; self-paced walking for 20 min with adjustments at 5, 10, or 15 min	The FS was administered every 5 min during the experimental trials	Outdoor walking resulted in more pleasure than treadmill walking, and pleasure decreased over time in both conditions	Indoor vs. outdoor exercise; Green exercise
Deutsch et al. (53)	*N* = 15; 10 male, 5 female; *M* age 55.4 ± 14.3 years; chronic phase post-stroke and ability to walk 100 feet without assistance and the ability to stand unsupported for 3 min	Within-subjects; Video games; Activities involved stepping and marching, two conditions self-paced, and two conditions game paced	Enjoyment measured with the PACES after completing each activity	No differences in enjoyment between conditions	Gamification; Video games
Dyrlund and Winninger (54)	*N* = 200; 126 female, 74 male; *M* age 20.69 ± 4.41 years; university students	Between-subjects; Music preference, Attentional focus; Treadmill walking at low, moderate, or high intensity for 20 min	The interest/enjoyment scale of the Intrinsic Motivation Inventory was used to measure enjoyment	There was no main effect or interaction effect for music or intensity on enjoyment	Music; Attentional focus
Elliott et al. (55)	*N* = 18; 8 males, *M* age 21.2 ± 0.9 years; 10 females, *M* age 20.7 ± 1.1 years; undergraduate students	Within-subjects; Music; Cycle ergometer exercise at 60–80% age predicted maximum HR	The FS was administered during exercise and responses were averaged across the bout	Pleasure was higher for motivational music compared to control, but not for motivational music compared to oudeterous (neutral)	Music
Emanuel et al. (56)	*N* = 19; 10 males, *M* age 31.6 ± 5.8 years; 9 females, *M* age 29.2 ± 3.4 years; resistance trained	Within-subjects; Number of repetitions, fixed or choice; Resistance exercise at 70% 1-repetition-maximum	Enjoyment was measured with a VAS	There was no difference between conditions	Self-selected vs. prescribed
Eston et al. (57)	*N* = 12; males; *M* age 20.8 ± 0.8 years; study 1	Within-subjects; Deception and expected duration; Treadmill exercise at 70% VO_2_ peak	The FS was administered during exercise	There was no main effect for conditions. Pleasure decreased between 9 and 10 min during the condition where participants expected to stop at 10 min but were asked to continue for another 10 min	Deception and expected duration
Eston et al. (57)	*N* = 8; males; *M* age 20.9 ± 0.8 years; study 2	Within-subjects; Deception and expected duration; Cycle ergometer exercise at 65% VO_2_ for 20 min	The FS was administered during exercise	There was no main effect for conditions. There was a significant interaction effect whereby pleasure decreased between 9 and 10 min during the condition where participants expected to stop at 10 min but were asked to continue for another 10 min. Pleasure was lower at 20 min for the unknown duration condition	Deception and expected duration
Feiss et al. (58)	*N* = 63; 32 males, 31 females; *M* age 25 ± 4.4 years	Between-subjects; Music tempo, fast tempo, slow tempo; Wall-sits and plank holds	The Affect Grid was used to measure pleasure-displeasure.	There was no effect of condition on pleasure-displeasure	Music; Music tempo
Feltz et al. (59)	*N* = 120; 60 females, 60 males; *M* age 19.41 ± 1.52 years; undergraduate students	Between-subjects; Exercise with “cyber buddy”; Plank for as long as possible	The PACES-8 was used to measure enjoyment.	There was no effect on enjoyment or exercise intentions	Social environment; Exercise partner
Focht (60)	*N* = 35; women; *M* age 22.14 ± 1.73 years; university students	Within-subjects; Indoor vs. outdoor exercise; walking at a self-selected pace for 10 min	The FS was administered before, during, and after exercise. A single-item measure was used to assess enjoyment postexercise.	Pleasure and enjoyment were higher for outdoor exercise	Exercise environment; Indoor vs. outdoor exercise
Focht and Hausenblas (61)	*N* = 30; women; *M* age 20.2 ± 1.65 years; *M* BMI 25.85 ± 3.6 kg/m^2^; high social physique anxiety, inactive women, university students	Between-subjects; public (university fitness centre) and private environment (nonmirrored laboratory); stationary cycling for 20 min at either a self-selected or imposed intensity (70%–80% age predicted max HR)	The FS was administered before, during, and after exercise.	Pleasure initially decreased during exercise in the public environment before rebounding by the end of the session. Compared to baseline, the public environment resulted in medium improvements during the final in-task measurement (*d* = .44) and large improvements postexercise (*d* = .86). Pleasure increased during exercise in the private setting with medium to large improvements compared to baseline (*d* = .55 to *d* = 1.23)	Social environment; public vs. private exercise
Frühauf et al. (62)	*N* = 14, 8 female, 6 male; *M* age 32.7 ± 10.8 years, *M* Beck Depression Inventory II score 17.9 ± 9.6; in-patients, mild-to-moderate depression	Within-subjects; indoor, outdoor exercise, group exercise; 60 min Nordic walking (outdoor) and cycle ergometer (indoor), RPE 11–14	The FS was administered before, every 15 min during, and after exercise.	No differences in FS score between outdoor exercise compared to indoor exercise and a sedentary control condition.	Indoor vs. outdoor exercise
Galway et al. (63)	*N* = 107; Functionality-focused group, 41 females, 10 males, *M* age 69 ± 6.28 years; Appearance-focused group, 40 females, 16 males, *M* age 69 ± 7.06 years	Between-subjects; Instructor focus, functional, appearance-focused cues; Group exercise classes of about 45 min	Enjoyment was measured using the PACES	There were no differences in enjoyment	Social environment; exercise instructor cues
Gillman and Bryan (64)	*N* = 78; 58 females, 20 males; *M* age 26.8 ± 6.62 years; insufficiently active adults	Between-subjects; Mindfulness, podcast, associative attentional focus; Walking at an intensity determined during a “Talk Test” for 30 min	The FS administered to capture how the participants “were feeling on average in the past (five/ten) minutes”.	Pleasure was higher in the mindfulness and distraction/podcast conditions compared to associative focus. Mindfulness and distraction/podcast were not different. The affective slope was also more positive in the mindfulness and distraction/podcast groups	Mindfulness
Glen et al. (65)	*N* = 20; 16 females, 4 males; *M* age 24.15 ± 5.9 years; recruited from university campus	Within-subjects; Exergaming, “track mode”, “game mode”; Cycling using a recumbent cycle ergometer at a self-selected intensity for 15 min	The FS was administered every 3 min during exercise. The PACES was used to measure enjoyment.	Pleasure and enjoyment were higher in the game mode compared to the track mode. Pleasure and enjoyment were higher in the track and game mode compared to control	Exergaming
Greenhouse-Tucknott et al. (66)	*N* = 20; males; *M* age 25 ± 4 years	Within-subjects; Prior exercise, completing a handgrip exercise task; Resistance exercise, submaximal knee extensor exercise	An amended FS was administered during the knee extension. The extremes were anchored based on the participants’ prior exercise experience (e.g., “most pleasant experience during previous physical activity”)	Participating in the prior handgrip task reduced pleasure experienced during the knee extension task	Other—Prior acute exercise
Haile et al. (67)	*N* = 32; males; Experimental group *M* age 22.6 ± 2.2 years; Control group *M* age 22.1 ± 2.3 years.	Mixed-design; imposed and self-selected exercise, one group was aware the intensities were matched; Cycle ergometer for 20 min	The FS was administered during and after exercise, and a mean was created for in-task responses. “Session Affective Response” was measured 15 min after exercise and seemed to function as a measure of remembered pleasure	Session affective response was greater than the in-task values for the self-selected exercise intensity, but not the imposed condition	Self-selected vs. prescribed; Autonomy
Hamlyn-Williams et al. (68)	*N* = 27; females; *M* age 14.6 ± 0.8 years; *M* BMI 20.9 ± 3.0 kg/m^2^	Within-subjects; Self-selected, prescribed intensity; Treadmill for 20 min	The FS was administered during exercise.	Affective responses were more positive in the self-selected condition compared to the prescribed condition	Self-paced vs. prescribed; Autonomy
Hanson and Buckworth (69)	*N* = 22; 11 men, 11 women; *M* age 26.6 ± 6.2 years; recreational runners	Within-subjects; Knowledge of the endpoint of exercise; Treadmill running for distance “based on their running history and average weekly or daily mileage"	The FS was administered at various times, between 1 and 4 min apart during exercise.	There was no effect of condition on affective valence.	Other; Endpoint knowledge
Hartman et al. (70)	*N* = 15; 13 men, 2 women; *M* age 23.4 ± 2.2 years; *M* VO_2_ max 46.0 ± 8.0 ml/kg/min	Within-subjects; Glycogen loaded; glycogen depleted; Cycling on a cycler ergometer at 10% above critical power until volitional exhaustion	The FS was administered 5 min before exercise and every minute during exercise.	Glycogen depletion resulted in a steeper decline in pleasure (*d* = .70)	Nutrition; Carbohydrate
Hawkins et al. (71)	*N* = 36; 9 active males, *M* age 26.67 ± 2.88 years; 9 active females, *M* age 24.89 ± 4.04 years; 8 insufficiently active males, *M* age 27.75 ± 7.57 years; 10 insufficiently active females, *M* age 28.70 ± 5.62 years	Mixed-design; Goal types, SMART, Open, Do-Your-Best; Six-minute walk tests.	The FS was administered before, during and after each six-minute walk test. Enjoyment was measured using the PACES at the end of each exercise session.	The SMART, Open, and Do-Your-Best conditions were more pleasant than the control condition. The active group also experienced more pleasure during the SMART, open, and DYB conditions. The open and DYB conditions were most pleasant for insufficiently active participants. Enjoyment was highest after Smart, open, and DYB conditions compared to control conditions. Active participants enjoyed the SMART condition more than the insufficiently active participants. Insufficiently active participants enjoyed the Open condition most.	Goal setting
Hobbins et al. (72)	*N* = 19; 3 females, 16 males; *M* age 33.4 ± 9.1 years; trained runners	Within-subjects; Normoxia, hypoxia; Treadmill running, HIIT protocol, RPE 16 for 4 min interval with 3 min recoveries	Remembered pleasure was measured using a VAS.	Pleasure was reduced in hypoxia	Other; oxygenation
Hu et al. (73)	*N* = 44; 22 male and 22 female; *M* age 14.27 ± 0.87 years; middle school students	Between-subjects; Self-efficacy feedback; Step exercise test, following prescribed pace until exhaustion	Enjoyment was measured using the PACES	Enjoyment was higher in the high self-efficacy group	Self-efficacy
Hu et al. (74)	*N* = 28; females; high-efficacy group *M* age 20.6 ± 2 years, low-efficacy group *M* age 20.1 ± 1.6 years; college students	Between-subjects; Self-efficacy feedback; Cycle ergometer max test followed by 30 min at 60% VO_2_peak	Enjoyment was measured using the PACES	Higher enjoyment of max test following false feedback to promote high self-efficacy. No difference after sub-max test when false information was reinforced prior to exercise	Self-efficacy
Hutchinson et al. (75)	*N* = 24; 14 male, 10 female; *M* age 21.3 ± 3.9 years; *M* VO_2_ max 53.82 ± 7.90 ml/kg/min	Within-subjects design; Music, music video; treadmill at 10% of max capacity above and below the ventilatory threshold	The FS was administered before, during, and after exercise. FS scores during exercise were averaged	Music videos were more pleasant regardless of intensity. Music was also more pleasant than control. Post-task FS scores for Music videos and music-only were higher than control during below ventilatory threshold exercise compared to pre-exercise. Post-task FS scores were higher than in-task FS scores for music videos and music-only.	Music; Video
Hutchinson et al. (76)	*N* = 24; females; *M* age 66 ± 8.5 years; participants in an exercise program for people with diabetes	Within-subjects design; Music, music video; Flexibility, aerobic, and resistance activities	The FS was administered during and after exercise. Enjoyment was measured with the PACES-8.	Affective valence was more positive in the music and the music-and-video conditions compared to control. Participants enjoyed the music-and-video condition most, compared to music only and control.	Music; Video
Hutchinson et al. (77)	*N* = 17; 9 male, 8 female; *M* age 28.1 ± 9.9 years;	Within-subjects; Music; Treadmill running set to an FS score of “+3 (good)” for 20 min	Remembered pleasure was measured using a VAS	The music condition resulted in greater remembered pleasure than the no-music condition, with large effects (*d* = .72).	Music
Hutchinson et al. (78)	*N* = 40 adults; 30 men, 9 women, 1 transgender; *M* age 35 ± 9.2 years	Within-subjects; Pattern of intensity, increasing, decreasing; Resistance exercise using a circuit of exercises, 55%, 65%, 75% 1RM	The FS administered during and after exercise. Enjoyment measured after exercise using the PACES-8. Remembered pleasure measured using the EVS Empirical Valence Scale and an ad-hoc measure ranging from 0 (most unpleasant) to 10 (most pleasant) sent via text message, 24 h after exercise.	Participants in the decreasing-intensity condition felt more pleasant throughout the exercise session and more pleasure following exercise. The decreasing-intensity condition also resulted in more enjoyment, and greater remembered pleasure shortly after and 24 h after exercising	Pattern of Intensity
Ijaz et al. (79)	*N* = 45; 28 male, 17 female; *M* age 26.7 ± 8.6 years	Between-subjects; Virtual reality, Static interface (picture of road), or Open World (moving Google Street View). “Trike” exercise, self-selected duration and intensity	Enjoyment measured using the interest/enjoyment subscale of the Intrinsic Motivation Inventory	Open World was more enjoyable than the static interface condition	Virtual reality
Jones and Ekkekakis (80)	*N* = 21; 16 women, 5 men; *M* age 34.67 ± 9.62 years; *M* VO_2_ Peak 29.14 ± 6.56 ml/kg/min among men, 22.67 ± 4.52 ml/kg/min among women; low active	Within-subjects; Immersion, head-mounted display (high immersion), television (low immersion), music videos; recumbent cycle ergometer for 15 min at the ventilatory threshold	The FS was administered before, during, and after exercise. Enjoyment was measured using the PACES.	Pleasure was highest during high immersion at all time points. Pleasure was maintained during low immersion and increased during high immersion. Pleasure declined during the control condition. High and low immersion were more enjoyable than control	Music; Video
Jones et al. (81)	*N* = 38; 19 women, 19 men; *M* age 21.1 ± 1.9 years; *M* VO_2_ max 43.91 ± 7.75 ml/kg/min	Within-subjects; Music, video (parkland scene), music and video; Cycle ergometer at 10% below and 5% above ventilatory threshold for 10 min	The FS was administered during and after exercise. Enjoyment was measured using the PACES.	Pleasure was higher during exercise for music-only and music-and-video condition compared to video-only and control regardless of intensity. Music-only and music and video conditions were more enjoyable that video only and control regardless of intensity	Music; Video
Jones et al. (82)	*N* = 18; 10 men, 8 women; *M* age 25.1 ± 5.1 years; *M* VO_2_ max 38.82 ± 10.73 ml/kg/min	Within-subjects; respite music, continuous music; Cycle ergometer HIIT protocol at 100% Watt max, 60 s intervals	The FS was administered during the last 15 s of each work and recovery bout. Enjoyment was measured with the PACES, remembered pleasure using a VAS.	There were no effects of condition on pleasure during HIIT protocol. Continuous music was more enjoyable (*d* = .66) and remembered as more pleasant (*d* = .5) than control; respite (played only during recovery periods) was not different from continuous or control	Music
Jones et al. (83)	*N* = 13; males; *M* age 20.2 ± 1.9 years, *M* BMI 21.7 ± 1.7 kg/m^2^; trained runners	Within-subjects; respite music, fast and slow tempo; Treadmill interval running at 20% difference between gas exchange threshold and VO_2_max, 5 min intervals	The FS administered prior to warm-up, and final 10s of each recovery bout (x5)	Fast-tempo music (125–135 bpm) during recovery period elicited higher FS scores	Music
Karageorghis and Jones (84)	*N* = 22; 11 women, *M* age 20.3 ± 1.6 years; 11 men, *M* age 19.6 ± 1.6 years; university students	Mixed-design; music tempo; treadmill at 40%, 50%, 60%, 70%, 80%, and 90% maximum heart rate reserve, 2 min duration	The FS administered 15s before the end of each condition.	There were lower FS scores in the no-music control conditions across all intensities compared to slow, medium, fast, and very fast tempi conditions. Medium-tempo music led to higher FS scores than the other music tempo conditions	Music
Karageorghis et al. (85)	*N* = 24; 12 women and 12 men; *M* age 22.5 ± 1.7 years	Within-subjects; Respite-active music, fast tempo, medium tempo; HIIT on a cycle ergometer, 60 s intervals at 100% Watt max	The FS administered 15s before end of each exercise bout and recovery period. Remembered pleasure measured with a VAS and enjoyment measured with the PACES.	Enjoyment was higher for medium tempo compared to control and fast conditions, and higher in the fast than the control condition; remembered pleasure was higher for medium tempo compared to control and higher for fast tempo compared to control. FS scores were lower in control compared to the medium and fast conditions during both exercise and recovery	Music
Karageorghis et al. (86)	*N* = 26; 13 women, 13 men; *M* age 18.9 ± 0.8 years; undergraduate students	Within-subjects; synchronous music, motivational, oudeterous; Circuit exercise to failure	The FS administered at the end of each circuit station. In-task FS responses averaged.	Females reported less negative affect than males in both music conditions. No main effect for music conditions compared to metronome control.	Music
Kendzierski and DeCarlo (87)	*N* = 44; 21 females, 23 males; age between 18 and 24; undergraduate students	Mixed-design; high and low boredom proneness, self-selected music; cycle ergometry at a “steady, comfortable pace” for 20 min	Enjoyment measured using the PACES	Enjoyment was higher when participants listened to music	Music
Kim et al. (88)	*N* = 60; Horseback riding simulator, young adults *M* age 28.8 ± 4.1 years, elderly adults *M* age 67.8 ± 5.1 years; Real horseback riding, young adults *M* age 20.5 ± 3.8 years, elderly adults *M* age 59.0 ± 3.0 years	Between-subjects; Simulated, actual horseback riding; Horseback riding at various speeds for 15 min	Enjoyment was measured using a VAS	Real horseback riding was rated as more enjoyable	Other; Horseback riding: Real vs. simulated
Krinski et al. (89)	*N* = 38; women; *M* age 45.64 ± 8.63 years; *M* BMI 35.12 ± 3.42 kg/m^2^.	Within-subjects; Indoor vs. outdoor exercise; Walking at a self-selected pace for 30 min	The FS was administered every 5 min during exercise. The PACES was used to measure enjoyment	Affective valence was more positive during outdoor exercise at minutes 15, 20, 25, and 30 during exercise. Greater enjoyment reported after outdoor exercise.	Exercise environment; Green exercise; Indoor vs. outdoor exercise
Kwan et al. (90)	*N* = 98; 59 women and 39 men; age 24.89 ± 5.98; *M* BMI 23.39 ± 4.03 kg/m^2^	Between-subjects; Anticipated affect, positive, negative; Treadmill exercise at an intensity just below the ventilatory threshold for 30 min	The FS administered before, during, and after exercise. Remembered pleasure was assessed	No significant effect on FS scores at 10 min, but significant difference at 20 min. Difference between experienced and anticipated affect was greater for negative anticipated affect compared to control. No effects on remembered pleasure	Other—Anticipated affect
Kwon et al. (91)	*N* = 20; 10 men, 10 women; age 22.5 (no *SD* provided); university students	Within-subjects; aromatherapy; Treadmill exercise for 30 min at 70–80% of maximum heart rate	The FS administered before, during, after exercise.	No inferential statistical analyses were performed (averages were reported in a figure). The authors concluded that aromatherapy increased pleasure during exercise and that pleasure was maintained after exercising	Aromatherapy
Legrand et al. (92)	*N* = 131; 42 women, *M* age 18.9 ± 1.1 years; 89 men, *M* age 19.5 ± 1.3 years; regular exercisers; university students	Between-subjects; Virtual reality, self-selected, imposed; Cycling or jogging for 10 min below ventilatory threshold	The Self-Assessment Manikin was administered during exercise	Self-selected interactive virtual reality exercise resulted in greater pleasure	Virtual reality; Autonomy
Lim et al. (93)	*N* = 23; men; *M* age 22 ± 4 years	Within-subjects; music, synchronous, asynchronous; cycle ergometer bouts at 90% of the ventilatory threshold for 6 min	The FS was administered during exercise	Music conditions were more pleasant than the no-music control and metronome conditions. Synchronous and asynchronous music conditions were not different	Music
Malik et al. (94)	*N* = 16; 8 boys, *M* age 12.4 ± 0.7 years, *M* daily MVPA 41 ± 16 min; 8 girls, *M* age 12.6 ± 0.8 years, *M* daily MVPA 32 ± 14 min.	Within-subjects; Pattern of intensity, increased, decreased, constant; High-intensity interval exercise on a cycle ergometer, eight 1 min bouts between 70% and 100% peak power	The FS was administered before, during, and after exercise. Enjoyment was measured using the Exercise Enjoyment Scale and PACES during exercise.	FS scores were lower during intervals 1–3 for decreasing intensity compared to increased and constant, but higher during intervals 7–8. EES data showed lower scores during intervals 1–2 for the decreasing intensity condition, but higher scores at interval 8. No effect of condition on PACES scores.	Pattern of Intensity
McAuley et al. (95)	*N* = 46; females; *M* age 20.4 ± 2.84 years; low-to-moderate activity; college students	Between-subjects; Self-efficacy feedback; 20 min of “moderate to hard intensity” using a Stairmaster exercise machine	The FS was administered every 2 min during exercise	No differences in FS scores between conditions	Self-efficacy
McDonough et al. (96)	*N* = 47; 25 female, 22 male; *M* age 11.8 ± 1.3 years; 83% black	Within-subjects; Exergaming, small and large group; Exergame (Xbox One Kinect Just Dance 2018) for 15 min	Enjoyment was measured using a 5-item questionnaire, administered immediately after each exergaming session.	Exergaming in small groups was significantly more enjoyable (*d* = .37)	Exergaming
McDonough et al. (97)	*N* = 20; 18 females, 2 males; *M* age 27.3 ± 4.3 years; retired Olympic athletes	Within-subjects; Exergaming, single and double players; Exergame (Xbox 360 Reflex Ridge) for 20 min	Enjoyment was measured using the Enjoyment and Self-Efficacy Scale, with separate means calculated for enjoyment and self-efficacy.	There was no difference in enjoyment between conditions	Exergaming
Miller et al. (98)	*N* = 25; 14 male, *M* age 20.3 ± 1.4 years; 11 female, *M* age 20.3 ± 1.4 years; college students	Within-subjects; Audiovisual, self-selected video, self-selected music; Recumbent cycling for 30 min at “an intensity as if they were exercising for fitness”	The FS was administered before, during, and after exercise	Affective valence was greater in the video and music conditions	Distraction; attentional focus; music; video
Minniti et al. (99)	*N* = 8; male; *M* age 25 ± 5 years; *M* VO_2_ max 53.7 ± 4.7 ml/kg/min; college runners	Within-subjects; Cooling collar; Treadmill running for 90 min in hot conditions, including 75 min at 60% VO_2_ max and 15 min all-out time trial	The FS was administered every 15 min during exercise	For steady-state section (75 min), pleasure was significantly higher in the no collar condition compared to the uncooled collar (*d* = .9) and cold collar (*d* = .2). No difference during the all-out section	Other; Clothing
Monedero et al. (100)	*N* = 34; 18 male, 16 female; *M* age 25.5 ± 6.9 years; *M* VO2peak 64.6 ± 9.9 ml/kg/min.	Within-subjects; Interactive video game; Cycling exercise at 55% peak power output for 30 min	Enjoyment was measured using the PACES after 10, 20, and 30 min of each trial. The PACES was modified using only 6 of the 18 original statements	Enjoyment was greater during the interactive cycling video game compared to conventional stationary cycling	Interaction; Video Games
Murray et al. (101)	*N* = 62; females; *M* age 20.2 ± 2.73 years; university students; analyses of 60 participants	Between-subjects; virtual reality, individual, companion; A rowing ergometer for 9 min	The FS administered during exercise. Enjoyment was measured with the PACES	There were no differences between groups in FS responses. Great enjoyment in individual and companion VR compared to control	Virtual reality; social environment
Neumann and Piercy (102)	*N* = 21; 12 female, 9 male; *M* age 24.52 ± 11.27 years; university students	Within-subjects; Attentional focus, distance focus, movement focus, breathing focus; Treadmill running at 70% VO_2_ max for 6 min blocks	Enjoyment was measured with the PACES after each block of exercise	There was no effect of condition on enjoyment	Attentional Focus
Neumann and Moffitt (103)	*N* = 40; virtual reality group 13 females, 11 males; *M* age 24.58; neutral images group 8 females, 8 males; *M* age: 24.37; university students	Between-subjects; Virtual reality, virtual runners, neutral images; Treadmill running at 70% Vmax (not VO_2_ max) for 21 min	The FS administered before, during, and after exercise. Enjoyment was measured using the PACES	There was no effect of condition on affective valence. Enjoyment was higher for the neutral images group compared to the virtual reality group, with large effects (*d* = .70)	Virtual Reality
Niedermeier et al. (104)	*N* = 42; 20 (48% female), *M* age 32 years	Within-subjects; Indoor vs. outdoor exercise; approximately three hours for each condition	The FS administered before, during, and after the conditions	FS scores increased from baseline in outdoor hiking compared to control. FS scores were higher for outdoor hiking compared to indoor treadmill from baseline and other timepoints (except rest period)	Exercise environment; Indoor vs. outdoor exercise
Oliveira et al. (105)	*N* = 18; men; *M* age 31 ± 7 years.	Within-subjects design; Self-selected and imposed intensity, virtual cycling; self-selected intensity and duration on cycle ergometer or matched	The FS was administered before, during, and after exercise. Enjoyment was measured using the PACES	There were no effects of condition for affective valence or enjoyment	Self-selected vs. imposed
Overstreet et al. (106)	*N* = 43; 28 women, 15 men; *M* age 19 ± 2 years; college students	Within-subjects; Television watching during exercise; Cycle ergometer at 40% peak oxygen consumption for 20 min	The FS administered during exercise. Enjoyment was measured using the PACES	Enjoyment was higher for the television watching condition. No differences in FS scores between conditions	Audiovisual; Television
Peacock et al. (107)	*N* = 12; men; *M* age 26 ± 5 years; *M* VO_2_ max: 54.9 ± 5.9 ml/kg/min	Within-subjects; Water, carbohydrate-electrolyte solution *ad libitum* during exercise; 20 min intervals on a treadmill, cross trainer, and cycle ergometer equivalent to 75% of maximum heart rate	The FS was administered at baseline, during, and after exercise	Pleasure was stable in the carbohydrate-electrolyte condition but declined in the water and no fluid trials. Pleasure was greater in the carbohydrate-electrolyte condition than the water condition (*d* = 1.0) and the no fluid condition (*d* = 1.6).	Nutrition
Peacock et al. (108)	*N* = 10; men; *M* age 25 ± 4 years; *M* VO_2_max 55.1 ± 8.6 ml/kg/min	Within-subjects; Water, carbohydrate-electrolyte solution *ad libitum* during exercise; aerobic (60 min) and resistance exercise (20 min) at self-selected intensity	The FS was administered at baseline, during, and after exercise.	The carbohydrate-electrolyte condition resulted in more pleasure at 40 min, 100 min, and 110 min during exercise	Nutrition
Philippen et al. (109)	*N* = 34; 18 women, 16 men; *M* age: 21.91 ± 2.55 years; university students	Within-subjects; Facial expression, smiling, frowning; Cycling ergometer exercise at 50%–60% of maximal heart rate reserve	The FS was administered and means for each condition were reported.	Participants reported more positive affective valence when smiling	Other; Facial expression
Plante et al. (110)	*N* = 112; 65 female, 47 male; no age data; college students	Between-subjects; Outdoor walk, indoor walk with virtual reality; indoor treadmill, outdoor college campus walk for 20 min, brisk walking pace	The Activation-Deactivation Adjective checklist was administered before and after exercise. Enjoyment was measured using the PACES	Exercising outside was more enjoyable and resulted in greater energy. Tension was lowest in the virtual reality and exercise condition	Video; Indoor vs. outdoor exercise, virtual reality
Plante et al. (111)	*N* = 128; female; *M* age 18.54 ± 1.02 years; university students; Study 1	Between-subjects; exercising alone, with a stranger, or with a friend; Cycle ergometer for 20 min at intensity between 60% and 70% max HR	PACES was administered to measure enjoyment	No differences in PACES scores	Social environment; Workout partner
Plante et al. (111)	*N* = 88; females; *M* age 19.31 ± 0.94 years; university students; Study 2	Between-subjects; exercising alone, alongside a friend, indoors, outdoors; indoor treadmill, outside campus walk for 20 min at 120–140 bpm (HR)	PACES was administered to measure enjoyment	Greater enjoyment when exercising outside with a friend and without a friend, compared to indoor exercise	Social environment; Workout partner
Plante et al. (112)	*N* = 128; 59 males, 69 females; *M* age 18.64 ± 1.3 years; university students; Study 1	Between-subjects; music, other people; Cycle ergometer at 70% age predicted maxHR for 20 min.	PACES was administered to measure enjoyment	Higher PACES scores when exercising with a friend they felt comfortable with. No effects of self-selected music on enjoyment	Music, outdoor exercise, exercise partner
Plante et al. (112)	*N* = 101; 61 female, 40 males; *M* age 18.84 ± 0.24 years; university students; Study 2	Between-subjects; outdoor exercise, music, other people; outdoor campus walk for 20 min at 70% age predicted maxHR.	PACES was administered to measure enjoyment	No differences in PACES scores between listening to self-selected music, walking with a friend, or control. Data combined with Study 1 to indicate greater enjoyment for outdoor exercise compared to indoor	Music, outdoor exercise, exercise partner
Pottratz et al. (113)	*N* = 28; females; *M* age 22.6 ± 3.3 years; Study 1	Within-subjects; Primes, music video, music; 8 min of treadmill walking at 65% heart rate reserve	The FS was administered during exercise. Remembered pleasure (VAS) and forecasted pleasure (EVS) measured after exercise	The music video + primes condition resulted in more pleasure than the music video and control conditions, but not more pleasure than the music condition. Remembered pleasure was higher in the music video + primes condition than in the music, music and video, and control conditions. Remembered pleasure was also higher in the music condition compared to control, and higher in music video compared to control. Forecasted pleasure was higher in the music video + primes condition than the music and control conditions, and in the music compared to the control condition, and the music video compared to control condition	Audiovisual; Subliminal Primes; music video
Pottratz et al. (113)	*N* = 24; 21 female, 3 male, *M* age 38.4 ± 7.2 years; Study 2	Within-subjects; Primes, music video; 10 min walking at a moderate intensity	The FS was administered during exercise. Remembered pleasure (VAS) and forecasted pleasure (EVS) measured after exercise. Enjoyment was also measured using the PACES-8	In-task affective valence was higher in the music video + primes condition than music video condition. Enjoyment, remembered pleasure, and forecasted pleasure was also higher in the music video + primes condition compared to the music video	Audiovisual; Subliminal Primes; music video
Privitera et al. (114)	*N* = 84; 52 women, 32 men; *M* age 19.8 ± 0.9 years; university students	Between-subjects; Television, enjoyable, not enjoyable TV show; Treadmill walking at 3.6 miles per hour for 10 min	Pleasure-displeasure was assessed with the Affect Grid, administered before and after exercise	Enjoyable TV while exercising led to highest pleasure compared to other conditions	Audiovisual; Television
Qin et al. (115)	*N* = 10; males; *M* age 27.3 ± 1.4 years); regular runners	Within-subjects; Carbohydrate, protein ingestion; Treadmill exercise at 70% of VO_2_ max for 90 min	The FS was administered at baseline and every 15 min during exercise	Higher FS scores in the carbohydrate + alpha-lactalbumin (protein) condition at 90 min, compared to control, and carbohydrate + whey (protein) conditions	Nutrition
Rhodewalt et al. (116)	*N* = 25; 12 women, 13 men; *M* age 22 ± 2 years; recreationally active	Within-subjects; Fed, fasted state; Treadmill exercise for 30 min at an intensity corresponding to a RPE of 13	The FS was administered at baseline, during, and after exercise. Enjoyment was measured using the PACES	There was no main effect of condition on affective valence or enjoyment	Nutrition
Rogerson et al. (117)	*N* = 24; 19 female, 5 male; *M* age 35.1 ± 20.1 years	Within-subjects; Green (outdoor) exercise, indoor exercise; Cycling exercise using a stationary cycle ergometer for 15 min at 50% heart rate reserve	Enjoyment was measured after exercise using a VAS ranging from “not at all” to “very much”	Condition did not influence enjoyment or intentions	Green Exercise; Indoor vs. outdoor exercise
Rose and Parfitt (118)	*N* = 32; 17 sedentary females, *M* age 43.9 ± 9.7 years, *M* BMI 26.5 ± 3.4 kg/m^2^; 15 active females, *M* age 46.4 ± 10.6 years, *M* BMI 24.8 ± 2.7 kg/m^2^	Within-subjects; Prescribed, self-selected exercise intensity; Treadmill for 30 min at self-selected intensity or close to ventilatory threshold (prescribed)	The FS was administered pre-exercise, every 5 min during, then 5 and 10 min post-exercise	Active females reported higher FS for self-selected intensity when completed first. FS increased from 30 min to 5 min post for self-selected condition. In the prescribed condition, the increase from 30 min to 5 min post was greater when prescribed completed first	Self-selected vs. prescribed
Schubert et al. (119)	*N* = 14; 8 women, 6 men; *M* age 24.9 ± 4.4 years; *M* VO_2_ max 47.6 ± 8.2 ml/kg/min	Within-subjects; Caffeine; Cycle ergometer exercise 65% VO_2_max for 60 min	The FS was administered every 15 min during exercise. Enjoyment was measured using the PACES	There was no difference in affective valence. Enjoyment higher in exercise with caffeine (*d* = .58)	Nutrition; caffeine
Shaver et al. (120)	*N* = 19; female; *M* age 26 ± 6.5 years; recreational runners	Within-subjects; Drinking, rinsing water; 15 km time trials, running outdoors	The FS administered before, during, and after running. The Activation-Deactivation Adjective Checklist was administered before and after each trial	The conditions made no difference in affective valence or energetic and tense arousal	Nutrition
Stanley and Cumming (121)	*N* = 88; 56 females, 32 males; *M* age 19.81 ± 1.48 years; physically active undergraduate students	Between-subjects; Imagery; Cycle ergometer at 50% of age-predicted HRR for 20 min.	The FS administered before, during, and after exercise. A single-item measure of enjoyment was administered during exercise	The enjoyment- and energy-imagery groups experienced more pleasure than the control group at minute 18. The energy-imagery group had more postexercise pleasure than the control group. The enjoyment- and energy-groups increased pleasure from pre- to post-exercise. All three imagery groups demonstrated more enjoyment than the control group at minute 18	Imagery
Stork et al. (122)	*N* = 20; 10 men and 10 women; *M* age 22.5 ± 4.3 years	Within-subjects; music; sprint interval exercise, four all-out 30 s Wingate tests	The FS administered before exercise, twice during recovery periods (one immediately after all-out exercise asking how they felt during the exercise, and the second how they felt at that time), and after exercise. Enjoyment was measured using an adapted version of the PACES	No effects of condition on affective valence during work or rest periods. Enjoyment was higher in the music condition than the non-music condition	Music
Stork et al. (123)	*N* = 24; 12 men and 12 women; *M* age 24.08 ± 4.61 years; low active	Within-subjects; music, podcasts; Low-volume sprint interval exercise, three all-out 20 s sprints on cycle ergometer	The FS administered at the end of each recovery interval and enjoyment measured using the PACES after each trial	Affective valence was more positive in the music condition compared to the no-audio control, and enjoyment was greater in the music condition than the podcast condition and no-audio condition	Audio; music
Swann et al. (124)	*N* = 78; 58 women, 20 men; *M* age: 55.88 ± 12.37 years	Mixed-design; Goal setting, open goals, do-your-best goals, SMART goals; six minute walk test	Enjoyment measured with PACES	Enjoyment was greater in the Open and SMART conditions than the control. The do-your-best goal was not different from other conditions	Goal setting
Tempest and Parfitt (125)	*N* = 14; 7 males, 7 females; *M* age 21.79 ± 2.15 years	Within-subjects; Imagery; recumbent cycle ergometer for 10 min at 5% above the ventilatory threshold, 10 min of passive recovery, then exercise at the respiratory compensation point until exhaustion.	FS was administered before, during, and after exercise	Affective valence declined less in the imagery condition compared to the control condition	Imagery
Toh et al. (126)	*N* = 12; males; *M* age 10.7 ± 1.2 years; *M* VO_2_ peak: 39.2 ± 3.8 ml/kg/min; *M* BMI 22.5 ± 1.8 kg/m^2^	Within-subjects; soccer games on different size courts; two halves of 15 min	Enjoyment was measured with the PACES (16-item) at the end of each game	There was no effect on enjoyment	Other; Pitch size
Tritter et al. (127)	*N* = 74; 32 male, 42 female; High efficacy group *M* age 20.9 ± 1.7 years; low efficacy group *M* age 21.8 ± 2.4 years; control group *M* age 22.6 ± 2.3 years; university students	Between-subjects; Self-efficacy feedback; Sprint interval training on a treadmill, four 30 s maximal sprints on a treadmill	Enjoyment was measured with the PACES after the cool down	The high self-efficacy group had higher enjoyment than the low-efficacy and control groups, with no difference between the control and low-efficacy groups	Self-efficacy
Turner and Stevinson (128)	*N* = 22; 14 male, 8 female; *M* age: 33 ± 8.3 years; runners	Within-subjects; Indoor, outdoor exercise; Self-paced running with the first 3 km at “steady-state pace” and the final 3 km “as fast as possible”.	The FS was administered before, during, and after exercise.	Affective valence increased from end-point of exercise to 10 min postexercise for the outdoor condition	Exercise environment; Indoor vs. outdoor exercise
van der Kooij et al. (129)	*N* = 28; 19 women, 9 men; *M* age 75.2 ± 6.6 years; community dwelling; experiment 1, analyses with 24 participants	Between-subjects; Gamification; Balance exercise for 4 min	Enjoyment assessed using the Interest/Enjoyment Scale from the Intrinsic Motivation Inventory and a “Quick Motivation Index” (*ad hoc*)	The gamified balance task was more enjoyable than the conventional balance task	Gamification
van der Kooij et al. (129)	N = 42; 23 females, 19 males; *M* age 24.5 ± 8.37 years; experiment 2	Between-subjects; Gamification, virtual environment to catch targets and avoid obstacles; Treadmill exercise at a comfortable speed for “about 30 min”	Enjoyment was assessed with the Quick Motivation Index after blocks of exercise	Enjoyment was higher in the gamified group	Gamification
Vazou-Ekkekakis and Ekkekakis (130)	*N* = 19; female; *M* age: 21 ± 2 years; *M* BMI 20.67 ± .02 kg/m^2^; sedentary university students	Within-subjects; Self-selected intensity, experimenter-controlled exercise intensity; Treadmill exercise with a matched intensity for 30 min	The FS was administered before and during exercise. AD ACL was administered before and after exercise. The Interest/enjoyment subscale of the Intrinsic Motivation Inventory was recorded after exercise	Increase in energetic arousal was greater in the autonomous condition. There was no effect on affective valence or tense arousal. The experimenter-controlled condition resulted in less enjoyment	Self-selected vs. prescribed; autonomy
Watson et al. (131)	*N* = 17; 14 male, 3 female; *M* age 15.1 ± 1.1 years; hockey players	Within-subjects; Self-selected or predetermined order of exercises; Resistance training, three sets of six repetitions	Enjoyment was measured with the PACES-8	The different order of exercises did not make any differences in enjoyment	Ordering of Exercise
Welch et al. (132)	*N* = 24; females; *M* age 23 ± 4.6 years; low active	Within-subjects; Expected duration, unknown duration; Cycle ergometer at 90% of ventilatory threshold for 30 min	The FS was administered before, during, and after exercise	Unknown duration resulted in declining pleasure from baseline to the last measurement during exercise	Expectations; Unknown exercise duration vs. known
White et al. (133)	*N* = 37; women; *M* age 50.11 ± 3.69 years; postmenopausal	Within-subjects; Simulated natural environments; Cycle ergometry for 15 min at a comfortable pace	The FS was administered before, during, and after exercise	The simulated urban environment resulted in less positive affect than the control (no images) lab environment. The first 5 min of exercise resulted in smaller changes in FS scores between green, blue, and urban images compared to control	Exercise environment; Simulated natural environments using video; Green, grey, blue exercise.
Williams et al. (134)	*N* = 59; 52 females, 7 males; *M* age: 47.7 ± 11.1 years; *M* BMI 31.9 ± 4 kg/m^2^; insufficiently active	Between-subjects; Self-paced exercise; walking at a moderate intensity or a self-paced intensity (but not to exceed 76% of max heart rate)	The FS was administered during and after exercise, and also randomly during the day (to control for baseline affective valence)	Self-paced exercise resulted in more positive affective responses, and affective responses predicted more exercise behaviour	Self-paced vs. prescribed
Williams and Raynor (135)	*N* = 29; women; *M* age 39.7 ± 12.3 years; *M* BMI 29.9 ± 6.6 kg/m^2^; sedentary or low-active women	Within-subjects; Self-selected intensity, imposed intensity; Treadmill walk at self-selected pace, experimenter set pace (same as self-selected pace), and experimenter set (20% faster than self-selected pace), for one-third mile	The FS administered before, during, and after exercise	There were no effects on affective valence	Self-selected vs. prescribed
Winchester et al. (136)	*N* = 10; men; *M* age: 21.8 ± 1.7 years; university students; moderately active	Within-subjects design; male, female observer; treadmill running at 60% of peak running speed for 20 min	The FS was administered during and after the exercise session	FS scores during exercise were higher in the male-observed and female-observed trials compared to the control (no observers) trial; post-exercise FS scores were higher in the female-observed condition compared to control. Observers were rated as “highly attractive”	Social Environment
Zeng et al. (137)	*N* = 12; 9 females, 3 males; *M* age 25.01 ± 4.74 years; *M* BMI = 22.84 ± 3.68 kg/m^2^; college students	Within-subjects; Virtual reality exercise cycling; Cycle ergometer between 65 and 85% age-predicted max HR for 20 min	Enjoyment was measured using a 5-item questionnaire, administered after exercise	Enjoyment was greater during virtual reality exercise than traditional stationary cycling (*d* = .89)	Virtual Reality; Exergaming
Zenko et al. (138)	*N* = 46; increasing intensity group 16 men, 6 women, *M* age 28 ± 5 years; decreasing intensity group 15 men and 9 women, *M* age 27 ± 4 years.	Between-subjects; Pattern of intensity, increasing, decreasing; Recumbent cycle ergometry between 0 W to W equivalent to 120% ventilatory threshold for 15 min	The FS was administered before, during, and after exercise. Remembered pleasure was measured with a VAS, and forecasted pleasure was measured using the EVS. Enjoyment was measured using the PACES	Decreasing intensity resulted in increasing pleasure during exercise, more postexercise pleasure, more remembered pleasure, more forecasted pleasure, and greater enjoyment	Pattern of Intensity
Zenko et al. (139)	*N* = 41; 26 women, 15 men; *M* age 32 ± 10 years	Within-subjects; Prompt to change intensity to maximize pleasure; cycle ergometer for 10 min at a self-selected pace	The FS was administered before, during, and after exercise. Enjoyment was measured with the PACES. Remembered Pleasure was measured with a VAS	Remembered pleasure was higher following the prompt condition. FS increased in prompt condition as time progressed compared to control. Enjoyment was higher in prompt condition	Affect-regulated exercise

FS, feeling scale; PACES, physical activity enjoyment scale. Measures of enjoyment, forecast, and remembered pleasure were taken after exercise unless otherwise stated.

### 3.1. Audio-visual strategies

Audio-visual strategies represent the largest proportion of studies in this review with music being the most frequently included strategy (33 out of 125 studies). Music has long been an adjunct to exercise but more recently other audio strategies such as podcasts have been tested experimentally.

#### 3.1.1. Music

One of the oldest studies included in this review focused on music and demonstrated the capacity to influence Feeling Scale [FS (140);] scores (which range from *very bad* to *very good*). Brownley et al. (40) reported that fast tempo music positively increased pleasure during low and moderate intensity treadmill exercise in 16 untrained participants. Music tempo has been a central feature of several studies [e.g., (20, 84)]. The study by Karageorghis and Jones (84) demonstrated that all music tempo conditions (slow [95–100 bpm], medium [115–120 bpm], fast [135–140 bpm], and very fast [155–160 bpm]) elicited greater pleasure than no music during treadmill exercise, and that medium tempo music elicited greater pleasure than the other music tempi conditions across intensities ranging from 40% to 90% max heart rate reserve. Music tempo has also been examined during interval training with results indicating limited effects of music *during* this mode of exercise [e.g., (82, 85)]. However, affective responses reported post-interval training have shown more consistent positive changes as a consequence of listening to music [e.g., (123)].

Beyond studies examining the internal components of music such as tempo, the effects of music have been examined more broadly. Campbell and White (43) reported greater enjoyment during a 20 min, moderate intensity treadmill walking task when listening to music in a study of 148 undergraduate students. Elliott et al. (55) found that “motivational” music led to greater pleasure than not listening to music during cycle ergometry at 60%–80% maximum heart rate. In comparison to no-music, and a tempo-matched metronome condition, Lim et al. (93) found that music applied synchronously and asynchronously was reported as more pleasant. Karageorghis et al. (86) reported that females recorded less negative affect than males during circuit training tasks while listening to music that was either motivational or motivationally neutral. Bigliassi et al. (33) compared music to a podcast condition and a no-audio control condition while participants walked a self-selected pace on a 400 m outdoor athletic track. They found the music condition led to the highest in-task FS scores and post-exercise Physical Activity Enjoyment Scale (PACES) (87) scores, and the podcast was also more enjoyable than the control condition. Hutchinson et al. (77) combined self-selected music with affect-regulated exercise and reported that participants found the music condition more pleasant while exercising at a higher intensity (*d* = 1.12). Further, participants recalled the music session as more pleasant (*d* = .72).

While there are often positive affective responses reported in music and exercise studies, some studies suggest this effect is not universal. Dyrlund and Winninger (54) tested the effects of most-preferred or least-preferred music on enjoyment of treadmill walking at a range of exercise intensities but found no differences with 200 university students. Chen et al. (44) examined the effects of listening to music compared to no-music in a small sample (*n* = 12) of participants with Down's Syndrome and reported no differences in enjoyment following treadmill walking. Feiss et al. (58) reported no effects on pleasure–displeasure when listening to fast or slow tempo music compared to a control condition for isometric tasks (e.g., wall sit).

#### 3.1.2. Music and video

Music has been employed as an extrinsic strategy alongside, and in comparison to, other strategies. Jones et al. (81) showed that music as a solo strategy and alongside a point-of-view video travelling through parkland was more pleasant than the no stimuli and the video-only condition during exercise above and below the ventilatory threshold. The 2015 study by Hutchinson et al. (75) showed a slightly different pattern of results with music videos resulting in the most positive affective responses during treadmill exercise, although music by itself was also more pleasant and enjoyable than a control condition. Bird et al. (34) demonstrated a similar pattern of results with pleasure reported as higher for music video conditions compared to music-only, and with both experimental conditions more pleasant than control. Hutchinson et al. (76) extended the music video experiments into a clinical context with diabetic patients and found that music video and music-only were effective at positively enhancing pleasure and enjoyment during flexibility, aerobic, and resistance exercise. Miller et al. (98) demonstrated utility for music and video as independent strategies to enhance pleasure during recumbent cycle ergometer compared to no stimuli in a sample of 25 college students. In 2019, Jones and Ekkekakis (80) examined the effects of music videos delivered using immersive head-mounted displays (i.e., Samsung Gear VR) compared to a wall-mounted television and a control condition. In their study, 21 adults with overweight and low fitness reported highest FS scores in the highly immersive music video condition during 20 min of recumbent cycle ergometry. Similarly, Bird et al. (36) reported a 360° video accompanied by music elicited the most pleasure and enjoyment in 18 university students during a 10 min cycle ergometry task.

#### 3.1.3. Virtual reality

The delivery of virtual reality (VR) stimuli has only recently shifted to head-mounted displays and some studies have examined virtual reality using screen projections [e.g., (29)]. In this section, virtual reality relates to computer generated simulations of avatars or environments. A study adopting the projection approach with 109 children with overweight did not find any significant differences in enjoyment in comparisons between screen-projected VR and a no-VR condition following treadmill exercise (29). Similarly, Neumann and Moffitt (103) did not find any differences in a screen-projected VR study examining the effects of running with virtual runners or neutral images. Contrastingly, Zeng et al. (137) found greater enjoyment during a VR condition delivered via a head-mounted display compared to a no-VR condition during a 20 min cycle ergometry task with 12 college students. Bird et al. (35) combined virtual reality cycling with music and found more pleasure and enjoyment for the VR, and VR with music conditions compared to control conditions in a sample of 24 undergraduate students. Also using a head-mounted display, Ijaz et al. (79) showed that an “Open World” condition affording participants an opportunity to move through Google Maps Street View images in time with the cycling cadence was more enjoyable than watching static images from the same platform.

### 3.2. Indoor and outdoor exercise

Examining differences between indoor and outdoor exercise is a popular approach for researchers, with different designs being employed to understand how the outdoors can influence feelings during exercise. A similar pattern of results has been shown in a number of studies with outdoor exercise being reported as more pleasant and/or enjoyable than indoor exercise (41, 52, 60, 89, 104, 128). The participants varied from physically active university students (60) to women over 40 with obesity (89) but the pattern of results was similar. However, Rogerson et al. (117) did not find any differences in enjoyment when asking participants to cycle on an ergometer placed in a field at a university campus, and cycle ergometry in a visually sterile laboratory. Similarly, Frühauf and colleagues (62) found no differences between indoor and outdoor exercise in a small group of in-patients with mild-to-moderate depression. Frühauf et al's study was one of few that included a clinical population but appeared underpowered to find statistical significance given the effect sizes (indoor exercise, *d* = 1.44; outdoor exercise, *d* = .64).

Studies have also examined the efficacy of digital representations of outdoor environments. Calogiuiri et al. (42) examined the effects of exercising outdoors compared to indoor exercise accompanied by virtual footage of outdoors and found that outdoor exercise was more enjoyable. White et al. (133) compared simulations of outdoor environments while cycling on a stationary bike for 15 min in a sample of 37 post-menopausal women; findings showed that green and blue exercise environments (e.g., countryside or coastal setting) were more pleasant than a control condition. Rather than comparing indoor and outdoor exercise, Crust et al. (51) found that a countryside walk was more enjoyable than walking in a green urban environment for a sample from British recreational countryside walking groups.

### 3.3. Nutrition

Studies including nutrition strategies have often focused on caffeine or carbohydrate supplementation and indicate that supplementation can positively influence affective responses.

#### 3.3.1. Caffeine

Using a mixed design and 16 healthy active men (8 endurance trained), Astorino and colleagues (22) observed a positive effect of caffeine supplementation on affective responses during a 10 km time trial cycling performance. Similarly, in a study of 12 younger male endurance runners, Backhouse et al. (26) observed more positive affective responses when caffeine was used in prolonged cycling (90 min at 70% VO_2_ max). Schubert et al. (119) compared caffeine consumption to placebo during cycling at 65% VO_2_ max for 60 min and observed no difference in affective valence, measured with the FS every 15 min during exercise. However, enjoyment was enhanced with caffeine with moderate effects (Cohen's *d* = .58).

#### 3.3.2. Carbohydrate

Backhouse et al. (25) observed no effects of carbohydrate ingestion on pleasure measured before, during, and after cycling exercise in 17 young male soccer players; this was in contrast to earlier findings among 9 endurance trained males, in which carbohydrate consumption enhanced pleasure in response to prolonged cycling [120 min at 70% VO_2_ max (27)]. Both studies used the FS (140) to measure affective valence before, during, and after exercise and the reason for the conflicting results is unclear. In another study of 15 healthy individuals (no signs or symptoms of cardiovascular, renal, or metabolic disease), Hartman et al. (70) noted steeper declines in pleasure during high-intensity cycling exercise (10% above critical power) when exercisers were glycogen depleted, compared to glycogen loaded.

Peacock et al. (107) compared the effects of water vs. no fluid vs. a carbohydrate-electrolyte solution during 20-min intervals on a treadmill, cross trainer, and cycle ergometer. They noted large positive effects of the carbohydrate-electrolyte solution on pleasure compared to the water (Cohen's *d* = 1.0) and the no fluid conditions (Cohen's *d* = 1.6). Similar positive effects of ad-libitum carbohydrate-electrolyte solution were observed by Peacock et al. (108), in a study of younger recreationally active men. Qin et al. (115) compared affective responses during exercise between four conditions (two with carbohydrate and protein ingestion, carbohydrate ingestion only, and placebo) among 10 regular male runners during prolonged treadmill exercise (90 min at 70% VO_2_ max). Affective valence was measured throughout the exercise session but was significantly more positive at 90 min after ingestion of carbohydrate and protein ingestion.

Contrasting findings were provided by Rhodewalt et al. (116). Rhodewalt et al. (116) used the FS to measure affective valence before, during, and after exercise, and the PACES to measure enjoyment after exercise, Rhodewalt et al. (116) observed no effects of exercising in a fed vs. fasted state during 30 min of treadmill exercise at a moderate intensity (Rating of Perceived Exertion of 13).

#### 3.3.3. Other nutritional factors

Backhouse et al. (24) studied 15 young (mean age: 21 ± 0.5 years) and aerobically fit (VO_2_ max: 65.0 ± 1.2 ml/kg/min) men and found that pleasure was greater when allowing fluid replacement; fluid replacement resulted in greater postexercise pleasure, with large effect sizes, in response to a prolonged bout of treadmill exercise (90 min at 70% VO_2_ max). In another study focused on fluid, but this time mouth rinsing with either a pink or a clear non-caloric and artificially sweetened solution, Brown et al. (39) observed that mouth rinsing with a pink solution enhanced pleasure in response to running at a self-selected pace for 30 min (compared to a clear, non-caloric artificially sweetened solution). Shaver et al. (120) observed no differences in affective valence or energetic or tense arousal, measured with the FS or the Activation-Deactivation Adjective Checklist (141), between conditions with drinking or rinsing water. In this study, 19 recreationally active females ran 15 km time trials outdoors, while either drinking or rinsing water.

### 3.4. Pattern of exercise intensity

Researchers have also investigated the impact of the pattern of exercise intensity on affective responses. Zenko et al. (138) studied both men and women using a between-subjects design. Participants exercised for 15 min on a cycle ergometer and either increased intensity (from 0 Watts to 120% of the Watts corresponding to their ventilatory threshold) or decreased intensity (from 120% of the Watts corresponding to their ventilatory threshold to 0 Watts) and the FS was administered before, during, and after exercise. Remembered pleasure (i.e., a retrospective global evaluation of the session) was measuring using a bipolar visual analogue scale (VAS), and forecasted pleasure (i.e., predictions about how future repeated bouts would feel) was measured using the empirically spaced anchors from the Empirical Valence Scale (EVS) (141). Decreasing intensity resulted in more pleasure during and after exercise, more remembered pleasure, more positive forecasted pleasure, and more enjoyment. In a later pre-registered study, Hutchinson et al. (78) largely replicated these findings in resistance exercise. Using a within-subjects design, participants completed three resistance exercise circuits that either increased in load (from 55% to 65% to 75% of 1 repetition-maximum) or decreased in load (from 75% to 65% to 55% of 1 repetition-maximum). Affective valence was assessed using the FS and enjoyment was assessed using the PACES-8 (142). The EVS was used to assess remembered pleasure shortly after exercise and an ad-hoc measure was used to assess remembered pleasure 24 h after exercising, via text message. The decreasing intensity resulted in more pleasure during exercise, more enjoyment, and more remembered pleasure. Also see Hutchinson et al. (143) for a more recent study, not included in the systematic search.

Malik et al. (94) completed a within-subjects design, this time using high-intensity interval exercise on a cycle ergometer; 16 adolescent boys and girls performed exercise at an intensity that either increased, decreased, or stayed the same. Enjoyment of exercise was measured using the Exercise Enjoyment Scale (144) during the HIIT protocol, and PACES after exercise; the FS was used to assess affective valence before, during, and after exercise. Decreasing exercise intensity resulted in more pleasure and enjoyment toward the end of the session. However, there was no effect of the exercise condition on enjoyment when measured with PACES. In another interval study, Cortis et al. (46) used a within-subjects design in which 18 participants performed interval cycling exercise in either an “ascending”, “descending”, or “mixed pyramid” pattern. The ascending pattern increased in intensity from 50% to 75% to 100% of peak power output (PPO) across three 3 min work bouts, with 3 min recovery periods at 25% PPO. The descending pattern reversed this, with bouts decreasing in intensity from 100% of PPO to 75% to 50%. The “mixed pyramid” pattern, the initial workload was 75% PPO, then 100% PPO, and ended with 50% PPO. Enjoyment was assessed using the Exercise Enjoyment Scale and there was no difference between conditions.

Although Watson et al. (131) did not directly alter the pattern or *order of exercise intensity* or *training load*, they studied the effects of the *order of exercises*. Using a within-subjects design and 17 elite hockey players, participants completed resistance exercises in either a predetermined or self-selected order. The order of exercise made no difference on enjoyment.

### 3.5. Prescribed vs. self-selected exercise characteristics

Several researchers have investigated the effects of allowing participants to self-select aspects of their exercise protocol; this has often focused on allowing participants to choose their exercise intensity, rather than prescribing exercise intensity [e.g., (118)]. Importantly, not all studies controlled for intensity (i.e., sometimes intensity was not directly manipulated, but was different between groups or conditions as an indirect effect of the exercise prescription). Haile et al. (67) tested the effects of self-selected exercise intensity in a mixed design, where the experimental group was unaware that the imposed workload was identical to the self-selected workload and the control group was fully aware. Affective valence was measured using the FS during exercise and “session affective response” was measured 15 min after exercise and seemed to function as an indicator of remembered pleasure. There was no impact on mean affective responses during exercise or remembered pleasure between prescribed or self-selected workloads or between groups (aware vs. unaware). This finding is consistent with a later finding by Oliveira et al. (105), but somewhat conflicting with an earlier report by Vazou-Ekkekakis and Ekkekakis (130), who found that self-selected exercise intensity (vs. matched prescribed intensity) enhanced energetic arousal and interest/enjoyment, but not FS scores. Hamlyn-Williams et al. (68) did find that affective responses were enhanced during treadmill exercise at a self-selected intensity among 27 adolescent females, compared to prescribed intensity.

Other reports by Williams and colleagues (134, 135) were also mixed, with one study indicating that self-paced exercise resulted in more positive affective valence (134) and one study indicating that allowing sedentary and low-active women to self-select their own walking pace (vs. a matched pace set by the experimenter) did not impact affective valence (135). Zenko et al. (139) allowed participants to self-select their exercise intensity on a cycle ergometer, although there was an emphasis on pleasure. In the experimental condition of a within-subjects design, participants were prompted to choose an intensity that maximized their pleasure. Remembered pleasure and enjoyment was higher when participants were prompted to maximize their pleasure, and pleasure was more positive over time during exercise when compared to the control condition. However, there may have been demand effects in this study as the experimenters did not control for participant-experimenter interaction (i.e., the level of attention may have been a confounding factor). Future investigators should try to keep interaction between participants and experimenters more consistent.

Self-selected *intensity* was not the only characteristic investigated; Emanuel et al. (56) allowed participants to choose their number of repetitions in a resistance exercise context (choice of between 8 and 12 repetitions vs. a fixed 10 repetitions using a within-subject design) and found no difference in enjoyment.

### 3.6 Mindfulness

Researchers have studied the effects of mindfulness on the affective experience of exercise and this has also been paired with other factors [e.g., music (48)]. There have been mixed results; some studies report positive affective responses during exercise and increased enjoyment in response to mindfulness conditions (47), but other results indicate that although mindfulness increases forecasted pleasure, it makes no difference on affective responses during exercise (48). In the context of yoga, mindfulness has resulted in more pleasure (pre-to-post exercise) compared to a focus on changing one's appearance. Researchers also found that focusing on changing one's appearance resulted in lower remembered and forecasted pleasure compared to both mindfulness and yoga-poses conditions (50). Other researchers have found positive effects of both mindfulness and distraction (podcasts) among 78 insufficiently active walkers (64).

### 3.7. Self-Efficacy

Researchers have generally found that self-efficacy manipulations (e.g., providing false or true feedback after completing an exercise task) can positively impact affective outcomes, including enjoyment (74, 127). Although, Hu et al. (73) found that self-efficacy feedback influenced enjoyment only after a maximal exercise test (not sub-maximal intensity). Further, although McAuley et al. (95) found that participants reported more pleasure after self-efficacy inducing feedback, this was not statistically significant.

### 3.8. Social environment

The effects of the social environment in which exercise takes place has been explored by several researchers although the manipulations are highly variable and include a wide array of specific strategies (e.g., being observed by others, exercising in a private or public setting, exercising with a buddy). Plante et al. (111) found that exercising with a workout partner (either a stranger or a friend) did not make an impact on enjoyment when cycling on a stationary ergometer, although enjoyment was higher when walking around campus with a friend, compared to indoor exercise. The researchers noted that “exercising ‘alone’ was actually in a busy university fitness club facility” (p. 97), providing threats to the validity of the study.

Winchester et al. (136) found that affective valence was higher among men who ran at 60% of their peak running speed for 20 min when being observed by “highly attractive” (p. 217) observers compared to a control condition. Session affect was higher after being observed by the female-observer (vs. the control condition). Thus, exercising with an observer appeared to be beneficial for this small sample of 10 men. However, the “observers” acted as sham research assistants and spoke to the participants during exercise and did not solely watch.

Focht & Hausenblas (61) investigated the affective impact of exercising in a public environment (i.e., a university fitness center) vs. a private, non-mirrored laboratory setting. Pleasure initially decreased in the public environment but rebounded; compared to baseline, exercising in the public environment resulted in moderate improvements in pleasure during the last in-task FS measurement (*d* = .44) and large improvements postexercise (*d* = .86). Pleasure also increased in private settings, with medium-to-large effects (*d* = .55, *d* = 1.23).

Galway et al. (63) recently investigated the impact of function-vs. appearance-focused cues from exercise instructors on body image, enjoyment, and intentions to exercise in the context of group exercise classes lasting about 45 min; the researchers found no difference in enjoyment, measured with the PACES. Feltz et al. (59) found that planking with an exercise “buddy” for as long as possible did not make an impact on either exercise enjoyment or exercise intentions. Interestingly, other studies have included the effects of the presence of other people *at different locations*. Exercising with other people who were ostensibly exercising over the internet at different locations at a university made no difference on affective responses (101). In contrast, exercising in a competition against five computerized players appeared to be more enjoyable than a conventional stationary exercise session, although this may have been due to playing a game, rather than competing against other players (100).

### 3.9. Gamification and exergaming

Gamification has become an increasingly popular strategy to empirically examine. In two experiments, van der Kooij et al. (129) found that enjoyment was enhanced after gamified treadmill and balance exercise (i.e., allowing participants to virtually play a gardening game by using their feet and allowing participants to avoid virtual obstacles by changing their centre of mass). Researchers have found promising effects of video games among university students (65). Paired with virtual reality, exergaming has been found to increase enjoyment of moderate-to-vigorous intensity cycling among a sample of 12 college students, with large effects (*d* = .89) (137).

Among participants who have previously experienced a stroke, video games that included stepping and marching in self-paced or game-paced conditions did not seem to make a difference in enjoyment (53). McDonough et al. (96, 97) have provided some mixed results. Among 47 adolescents from minoritized backgrounds, exergaming (Just Dance 2018 on Xbox One Kinect) was more enjoyable when in a small group compared to a large group (96). In another within-subjects design, exergaming in single player or double player mode did not make an impact on enjoyment among 20 retired Olympic athletes (97). It is important to note that in both of these studies, exergaming was not compared to a control (i.e., exercising without gamification), indicating that the factor of exergaming itself was not manipulated.

### 3.10. Other strategies

Finally, several other extrinsic strategies have been manipulated by researchers although most of these have not been studied in more than a few studies. These factors include priming (19, 113), cognitive load (23), transcranial direct brain stimulation (28), facial expressions (37, 109), the color of screens around a treadmill (38), mirrors (45), deception and expected duration (57), prior bouts of exercise [i.e., handgrip tasks (66)], knowledge of the endpoint of exercise (69, 132), goal setting (71, 124), hypoxic environments (72), real vs. simulated horseback riding (88), manipulations of anticipated affect (90), aromatherapy (91), cooling collars (99), attentional focus (e.g., (102), imagery (121, 125), and pitch size (126). The variety of factors studied is an indication of intense interest in enhancing pleasure.

### 3.11. Quality assessment

**Figure 2:**
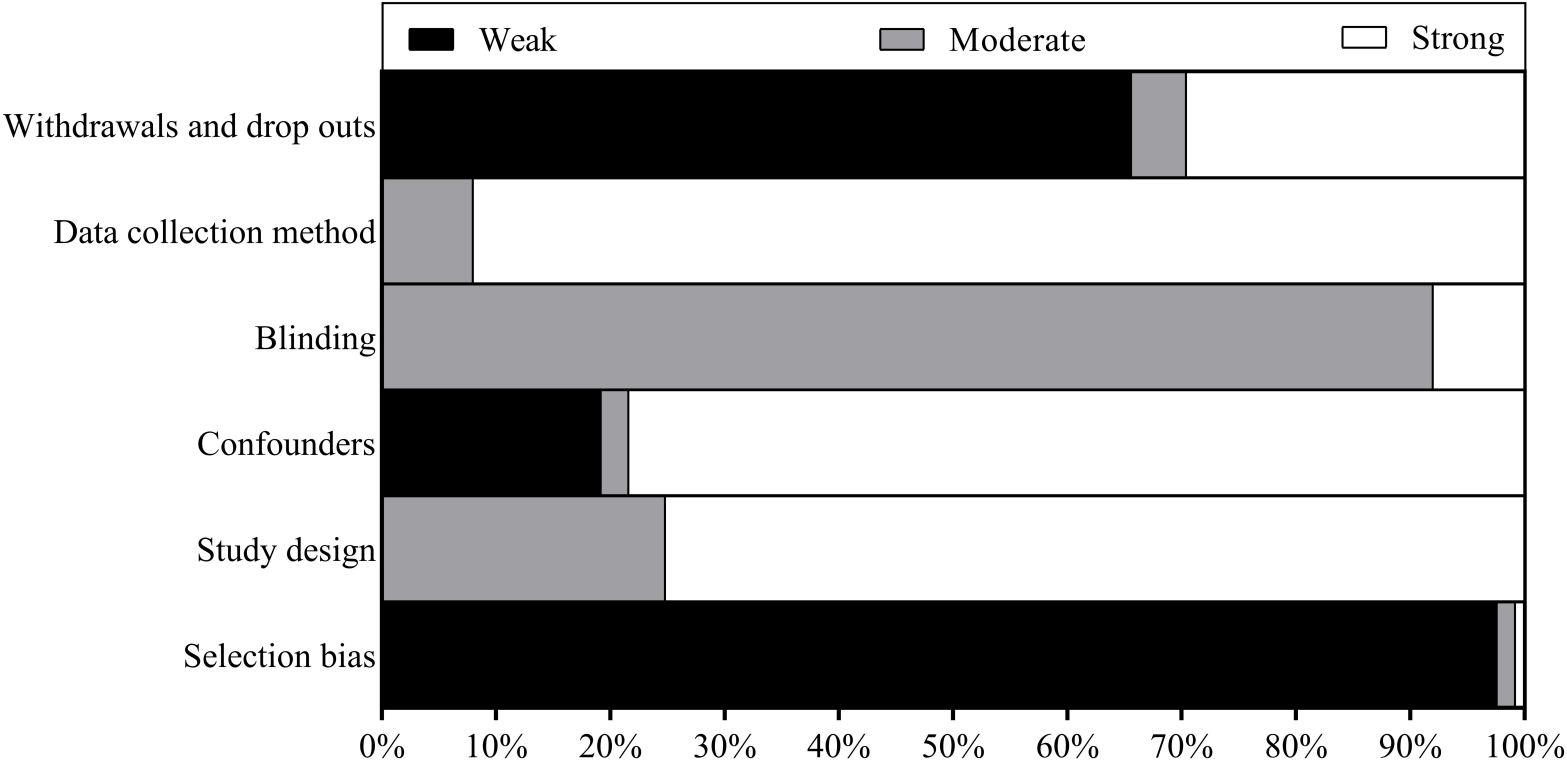
Quality assessment.

Of the 125 studies, 89 were categorised as Weak, 35 as Moderate, and 1 as Strong. Further details of the individual components that contributed to the global ratings are provided in Figure 2. In addition to the quality check, a check regarding *a priori* power calculations was conducted to assess the extent to which the number of participants recruited was appropriate. 72 of the 125 studies (58%) did not include an *a priori* power calculation for the sample size.

## 4. Discussion

This review aimed to collate studies that examined manipulations designed to influence affective responses during and immediately after exercise. 125 suitable studies were identified and they spanned a range of extrinsic strategies.

### 4.1. Extrinsic strategies

The body of evidence for listening to music during exercise appears broadly consistent and supportive of a capacity to positively influence pleasure. Music tempo appears to be a salient factor for consideration when deciding the music to be used. The majority of studies on this popular topic have been conducted with healthy, young adults and the consequence of this data for different populations are unknown. For example, Chen et alia's (44) study with participants with Down's syndrome revealed no difference in affective responses between music and no music conditions. This study appears underpowered but is demonstrative that further studies are needed to verify the effects of music during exercise in different populations. The use of video alongside music appears to confer an additive effect wherein affective responses are enhanced. Additional research on the specific types of video footage that are most beneficial is required, but initial work on music videos appears promising and Hutchinson et alia’s (76) work in a clinical population showed positive results. For virtual reality, the emergence of widely available head-mounted displays appears to have changed the findings for virtual reality studies. Those designs including head-mounted displays report more positive affective responses to the VR stimuli than was previously reported in screen-projected VR [e.g., (35)]. The greater immersion created by the head-mounted displays appears a relevant factor. The participants of the VR studies were typically young and active which limits to applicability of these findings to other groups.

Outdoor exercise appears to consistently show more positive affective responses than indoor exercise and this has been shown across different ages and activity levels. However, there appears a gap in research for work with clinical populations with only Frühauf et al. (62) exploring the strategy in a clinical sample. For some people, outdoor exercise is not easily achievable (particularly in green spaces) and initial attempts to create digital or virtual recreations of outdoor exercise have shown mixed results. Direct comparisons to outdoor exercise do not appear favourable for virtual green exercise (42), but when comparing to indoor exercise without virtual nature, the results are more positive (133). Additional work on virtual nature during exercise appears warranted given the incapacity of many people to access green spaces (145).

A strategy that has received recent increased attention is the pattern of exercise intensity. That is, does it matter whether an exercise session starts or ends with a high intensity, or higher levels of pleasure? The findings of Zenko et al. (138) and Hutchinson et al. (78, 143) aligned with earlier behavioral science research focused on the peak-, end-, and trend- rules, which indicate that overall summary evaluations are highly influenced by the most intense moment, last moment, and overall trend (e.g., increasing or decreasing pleasure) of an experience (146, 147). Further, these results are aligned with prior research from exercise psychology demonstrating that the peak- and end- of an experience explains a large percentage of the variance in one's overall memory of the experience (148).

Although not matching the inclusion criteria of this systematic review, a study by Stuntz et al. (149) found that, among collegiate athletes, increasing exertion resulted in more positive feeling states when increasing exertion resulted in more feelings of accomplishment. While it is clear that affective responses are sometimes meaningfully predictive of summary evaluations, including enjoyment, remembered pleasure, and forecasted pleasure, it is also clear that these summary evaluations are biased by memory and other cognitive filters and appraisals (9). Thus, there is need to further determine the conditions, contexts, and populations that may benefit from manipulating the pattern of exercise intensity. At present, it does not appear that the benefits are universal.

Lastly, it is important to note that several studies did not manipulate or control for intensity directly, and intensity may have differed between groups as an indirect effect of some extrinsic strategy [e.g., exergaming (97)]. As an example, for McDonough et al. (97), the physiological outcomes of exergaming alone or in pairs were relevant and therefore not standardized. However, there was greater energy expenditure and more steps when engaging with the exergame alone compared to with a partner. Since exercise intensity is a highly reliable determinant of affective responses (10, 11), one must acknowledge that any study that unintentionally included different intensities between groups or conditions may have contained an influential confound.

### 4.2. Quality check

The data presented in Figure 2 displays a mixed picture regarding the quality of studies. Beyond the global ratings of 71% Weak, 28% Moderate, and 1% Strong studies, 98% of studies were categorised as Weak for selection bias and this represents a significant issue for this field. A weak categorisation reflects self-referred participants, as opposed to random selection from a comprehensive list of individuals (Strong). Participants in the included studies would often be those who responded to adverts or be students at the authors' institution. These issues with selection bias limit the relevance of this field in contributing to wider discussions regarding exercise behaviour. However, if a study aims to influence affective responses of, for example, low active individuals with overweight from the general population, there is no list that participants can be drawn from. Therefore, there will be some instances where authors have made attempts to include representative participants, but the item used in the chosen quality check tool did not account for this.

The quality check results for study design and confounders indicated Strong in most studies. A key factor in the categorisation of Strong for Study Design was the use of randomisation for condition/group allocation. Many of the designs adopted a crossover RCT approach that we categorised as Strong. Following this, the effects of confounders was generally low as many studies included participants as their own controls. Moreover, in studies with separate groups, authors often demonstrated no significant differences between groups on important variables such as age.

The majority of studies were categorized as Moderate for Blinding as procedures were often not stated. The studies with Strong ratings were typically nutrition studies with effective placebo designs regarding drinks or caffeine. Many of the studies included in the review make blinding of the conditions difficult (e.g., a participant will know whether they are listening to music or not). However, researchers could make greater efforts to blind the true nature of the study and report their procedures for doing this. For example, Tritter et al. (127) provided participants with a letter stating the purpose of the study was to compare the effects of static or dynamic stretching, thereby attempting to hide the true nature of the study relating to affective responses to self-efficacy manipulations. We recommend that researchers make explicit statements to indicate whether outcome assessors were aware of the intervention or exposure status of participants and whether study participants were aware of the research questions. This would allow for more accurate assessment related to blinding.

A consequence of our inclusion/exclusion criteria was that we only included studies with certain pre-identified approaches to measurement. The majority of the studies in this design were categorised as having Strong data collection methods owing to the use of valid and reliable tools. The poor reporting of withdrawals and drop-outs led to two-thirds of studies categorised as Weak. The main drivers for 71% of studies receiving a global categorisation of Weak were issues with selection bias and poor reporting of withdrawals and dropout; one of these issues is easily remedied with improved reporting while the other is more challenging.

It seems common in exercise and sport science to not report dropouts and withdrawals unless participants dropped out or withdrew during a study. In other words, if zero participants dropped out or withdrew, it can often be assumed that the dropout and withdrawal rate was 0. However, according to the quality assessment tool used in this study, a weak rating must be assigned if the withdrawal and drop-out rates were not explicitly described. We recommend that researchers explicitly report dropout and withdrawal rates for all studies; this would also include explicit statements indicating if the follow-up and adherence rate was 100%. Overall, typical reporting practices might have inflated the number of “weak” and “moderate” quality ratings in this literature.

In addition to thorough and explicit reporting of the criteria highlighted in the quality assessment tool, the field would benefit from greater focus on important issues such as: *a priori* power analysis or sample size justification; clear research questions; preregistered methods; and theoretically and psychometrically sound measurement practices. Only 53 of the studies included in this review included some form of sample size justification, including *a priori power* analysis. Since underpowered studies may be less replicable than adequately powered studies, researchers must work to recruit more sufficient samples to ensure reliable effect sizes and conclusions. The percentage of studies with some level of sample size justification (about 42%) was higher than previously reported for sport and exercise science more broadly [i.e., only 22.67%; (150)]. Nonetheless, more than half of the studies included in this review had no sample size justification, indicating that this is an area for improvement.

### 4.3. Recommendations

A wide range of extrinsic strategies are presented in the review and provide practitioners and researchers with options for changing how people feel during, and after, exercise. However, the evidence supporting some of the strategies is weak in terms of quality of the study and also weak in terms of number of studies to support that evidence. Researchers have attempted many strategies to improve affective responses to exercise but several of these strategies have only been studied one or a few times (e.g., cognitive load, transcranial direct brain stimulation, facial expression, goal setting, manipulations of anticipated affect, imagery). The diverse array of research strategies, frequency of unjustified sample sizes, varying levels of research quality, and various research contexts highlights a need for replication attempts with many of these extrinsic strategies. In a recent study, for example, the findings of Pottratz et al. (113) were tested and failed to replicate (151). We speculate that this would not be a unique result if more replication attempts occurred; instead, more replication attempts in more research contexts (e.g., different cultures, ages, environments) might highlight highly variable effects.

The first practical recommendation is that new and innovative strategies are tested in robust research designs and with high quality reporting; this would include preregistration of study methods whenever possible (152) and full sample size justification (153). More high-quality design and reporting would also include stronger participant selection processes and transparent reporting of withdrawals and drop-outs. The second recommendation is for efforts to be directed towards replication of results especially of, but not limited to, strategies with one or two studies supporting their use (e.g., attentional focus, imagery). When choosing strategies for study, researchers should justify their choices and provide a rationale for why and how they believe the strategy will have an impact on affective responses. The third recommendation is to forge stronger links between practitioners and researchers to develop greater awareness of how strategies can be delivered outside of the laboratory in practical settings. The fourth recommendation is to conduct longitudinal studies examining the effectiveness of new strategies, and in those where only efficacy has been demonstrated (154). The fifth recommendation is for researchers and practitioners to engage with participants and clients in co-production of strategies [e.g., (155)] to help create meaningful and more positive experiences during exercise. The sixth recommendation is for studies to be conducted with participants in greater immediate need. The majority of studies included healthy, active participants but perhaps a significant impact this field could make is shaping the exercise experience for people who are overweight, obese, inactive, or among clinical populations. By changing experiences of people in those groups, our field might contribute to meaningful changes in positive outcomes with greater impact at individual and societal levels. Finally, the options for strategies to positively improve the exercise experience are limited only by our imagination. Sport and exercise psychologists already employ a number of strategies with their clients (e.g., self-talk, thought stopping) and these could be examined further with the aim of shaping how people feel during exercise. More broadly, a number of behaviour change techniques are employed in other health-related domains to positively change behaviour and these might also offer some direction for further experimental testing in an exercise context [see (156)]. As new technologies continue to develop (e.g., artificial intelligence), these offer avenues of exploration for researchers seeking to positively change how people feel during exercise.

### 4.4. Limitations

Many exercise trials are performed in the presence of additional potential extrinsic strategies although these are not captured within the study designs. For example, music might be the primary independent variable, but the researcher might also interact more with participants, and this could also be a source of affective influence during exercise. Further, there are studies that have combined strategies and the designs and analytical approaches do not always permit separation of the (possible) differential effects. These issues can limit the certainty of the results and the interpretations presented herein.

#### 4.4.1. Deviations from preregistration

While we did our best to adhere to the preregistered protocol in a good-faith manner, there were several deviations. We did not perform a backward or forward search of systematic reviews discovered in the initial search due to the large number of records initially retrieved and impracticality. We also performed multiple rounds systematic searches in order to be inclusive of more current literature. Finally, reasons for exclusion are not shown on the PRISMA diagram due to the exceedingly large number of reasons for exclusion (i.e., many articles excluded for multiple reasons).

## 5. Conclusion

We have reviewed numerous strategies and sought to focus on studies with high measurement quality. There are strategies that have been shown to positively influence how people feel during exercise and these provide options for practitioners to implement. From the included studies, music, music videos, immersive virtual reality, outdoor exercise, caffeine, high-to-low pattern of exercise intensity, self-selected exercise intensity, and manipulation of self-efficacy all offer implementable strategies to change how people feel during exercise. We have proposed several ways to improve the quality of studies in this field with the aim of enhancing the impact that these strategies might have.

## Data Availability

This systematic review did not include the collection of any original data.
